# Impact of residue accessible surface area on the prediction of protein secondary structures

**DOI:** 10.1186/1471-2105-9-357

**Published:** 2008-08-31

**Authors:** Amir Momen-Roknabadi, Mehdi Sadeghi, Hamid Pezeshk, Sayed-Amir Marashi

**Affiliations:** 1 Department of Biotechnology, College of Science, University of Tehran, Tehran, Iran; 2 National Institute of Genetic Engineering and Biotechnology, Tehran-Karaj Highway, Tehran, Iran; 3 Bioinformatics Group, School of Computer Science, Institute for Studies in Theoretical Physics and Mathematics (IPM), Niavaran Square, Tehran, Iran; 4 School of Mathematics, Statistics and Computer Sciences and Center of Excellence in Biomathematics, College of Science, University of Tehran, Tehran, Iran; 5 IMPRS-CBSC, Max Planck Institute for Molecular Genetics, Ihnestr. 63-73, D-14195 Berlin, Berlin, Germany; 6 DFG-Research Center Matheon, FB Mathematik und Informatik, Freie Universität Berlin, Arnimallee 6, D-14195 Berlin, Germany

## Abstract

**Background:**

The problem of accurate prediction of protein secondary structure continues to be one of the challenging problems in Bioinformatics. It has been previously suggested that amino acid relative solvent accessibility (RSA) might be an effective factor for increasing the accuracy of protein secondary structure prediction. Previous studies have either used a single constant threshold to classify residues into discrete classes (buries vs. exposed), or used the real-value predicted RSAs in their prediction method.

**Results:**

We studied the effect of applying different RSA threshold types (namely, fixed thresholds vs. residue-dependent thresholds) on a variety of secondary structure prediction methods. With the consideration of DSSP-assigned RSA values we realized that improvement in the accuracy of prediction strictly depends on the selected threshold(s). Furthermore, we showed that choosing a single threshold for all amino acids is not the best possible parameter. We therefore used residue-dependent thresholds and most of residues showed improvement in prediction. Next, we tried to consider predicted RSA values, since in the real-world problem, protein sequence is the only available information. We first predicted the RSA classes by RVP-net program and then used these data in our method. Using this approach, improvement in prediction was also obtained.

**Conclusion:**

The success of applying the RSA information on different secondary structure prediction methods suggest that prediction accuracy can be improved independent of prediction approaches. Thus, solvent accessibility can be considered as a rich source of information to help the improvement of these methods.

## Background

The problem of accurate prediction of protein three-dimensional structure continues to be one of the challenging problems in Bioinformatics. The large-scale genome sequencing efforts have made this problem even more significant. Roughly 50% of the proteins in a genome have at least one homolog in protein structure databases and their structure can be predicted efficiently by homology modeling [1,2]. However, for the other half of the sequences no structural template is currently known. To date, the performance of *ab initio *three dimensional prediction methods are still far from being perfect [3-5]. Therefore, in order to obtain information about the structure of a novel protein, one may consider simpler tasks, like one dimensional prediction of protein characteristics [6]. Acquiring such information is a key step in understanding the relationship between the protein folding and protein primary structure. The goal of protein secondary structure (SS) prediction methods is to predict whether each residue is in a helical structure (H), a strand (E), or in other structures (traditionally referred to as coil, C).

In the past decades, many prediction methods based on the database of known protein structures have been developed. Historically, the first generation of the SS prediction algorithms was developed by Chou and Fasman. [7,8] This algorithm, which is usually referred to as the Chou-Fasman method, tries to find structures based on the difference in the probability of observing each of the twenty residues in helices, sheets and other structures. This method has an accuracy of about 50–60% [7,8], although it has been shown that this method can be improved greatly with the application of several amendments [9]. It should be noted that other statistical methods (mainly based on hidden Markov models) have been also applied for protein SS prediction [10,11] and it seems that their prediction accuracies are comparable to current methods.

The second generation of SS prediction methods started by the method of Garnier, Osguthorpe and Robson (GOR method) [12] and improved in several steps [13]. This method, with an information theory approach, relates sequence to SS type and evaluates the state of each residue with a sliding window approach. Using this approach, better prediction accuracies, up to 64%, can be obtained [14].

The third generation methods use multiple sequence alignment and machine learning techniques like nearest neighbors and neural networks to predict the secondary structure. APSSP [15], JPred [16], SSpro [17], PHD [18], PSIpred [19], PMSVM [20], and other methods based on support vector machines [21-23] can be considered as the representatives of this generation. These methods generally achieve very good prediction accuracy, of up to 76%. It should be noted that recently, achievement of 80% accuracy is reported using a large-scale training [24].

Some years ago, it was thought that improvement of the methods will steadily result in the improvement of the SS prediction accuracy in the future [25], but now it seems that there is some kind of "barrier" that prevents all the above mentioned approaches to leave the 80% accuracy behind, and approach the theoretical prediction limit, which is estimated to be about 88% [26] or maybe up to 90–95% [27]. One possible barrier for SS prediction might lie in the neglect of other factors that may influence the tendencies of amino acids for being in different secondary structures. For example, it has been reported that amino acid propensities for secondary structures are influenced by the protein structural class [28,29], and by the organism from which the proteins are obtained [30].

It has been previously suggested that more accurate SS predictions can be achieved by taking relative solvent accessibility (RSA) into account [31-33]. The logic for the usefulness of such information lies in the fact that the environments around the protein residues can affect their propensities for different structures [34], and therefore, amino acids may behave differently when they are in the protein interior vs. surface of protein [35-39]. This effect is extensively studied in case of internal and surface beta-strands [40].

Based on these observations, one may ask why RSA is not routinely used today in the prediction of protein secondary structures. The answer lies in the fact that RSA prediction is not an easy task itself. The two original reports simply used DSSP [41] assignments to extract RSA information [32,33]. However, in the real-world version of the problem, protein sequence is almost always the only available information. For that reason, it was later tried to predict real-value RSAs [42,43] and to apply it for the improvement of protein SS prediction, in a method called SABLE [31]. While the performance of SABLE seems to be very good (i.e. 79.6% accuracy in CASP 6; see ), there seems to be much room for improvement of the method, as SABLE relies on an RSA prediction method with a correlation coefficient of 0.66 [31].

In the present work, we investigate the effect of the alteration of the RSA threshold on prediction accuracy. Our results imply that significant improvements in the prediction of SS can be obtained if the RSA cutoffs are selected according to the residues. We also discuss why predicted real-value RSAs might not be suitable for the improvement of SS prediction at this moment. Finally, we suggest that RSA prediction should be combined with the present SS prediction techniques, since the addition of RSA information improves the prediction, independent of the prediction approach.

## Results and discussion

### The effect of application of different RSA thresholds on the prediction of secondary structures

It was previously reported that when a 25% threshold for predicted RSA values is used to classify residues into {*B*, *Ex*} classes (i.e. Buried vs. Exposed; see Materials and Methods), this additional information increases the accuracy of SS prediction [31]. We decided to try other thresholds to see how they affect the predictions.

**Figure 1:**
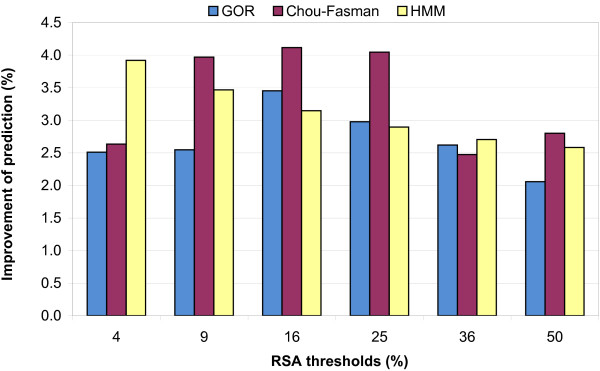
Percentage of improvement in secondary structure prediction accuracy by addition of RSA information for the GOR (A), Chou-Fasman (B) and HMM(C) methods using leave-one-out cross-validation and different thresholds in two-state classification of RSA.

In our analysis, we first investigated the effect of adding the actual RSA values (obtained from DSSP files), for different RSA thresholds using GOR, Chou-Fasman and HMM (Hidden Markov Method). Accuracies of SS prediction for GOR, Chou-Fasman and HMM methods, without consideration of RSA information are summarized in Additional file 1. Figure 1 depicts the level of improvement of SS prediction, compared to the prediction accuracy of classical method [see also Additional file 2, 3, 4]. For all selected thresholds, some improvements are obtained which is consistent with the results obtained by other investigators [32,33]. Our results suggest that the best threshold for the improvement of SS prediction in GOR and Chou-Fasman methods is about 16%, while HMM performs best with a 4% RSA threshold. Therefore, the 7% cutoff used by Zhu and Blundell [33], and also the 50% cutoff used by Macdonald and Johnson [32] might not be optimal.

As an additional test, we also divided amino acids into three discrete groups, i.e. we classified the residues to buried, intermediate and exposed, [35]. For each classification, therefore, a fixed threshold pair is used. The results for these methods are presented in the Additional file 5. The results generally show that classification into three groups yields a better result compared to a two-group classification. Among the tested classifications, namely [4%,16%], [9%,16%], [9%,36%] and [16%,36%], the first pair was the best choice for all methods.

**Figure 2:**
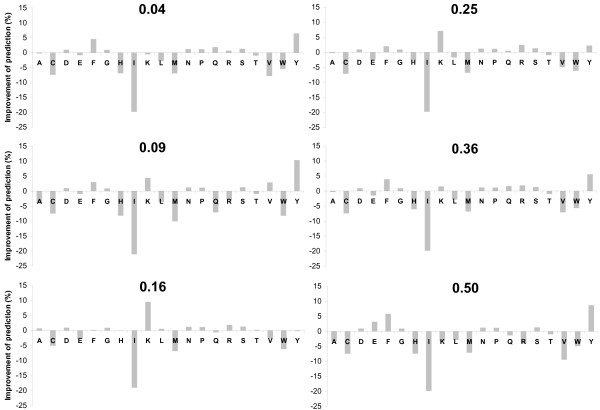
Percentage of improvement in secondary structure prediction accuracy by addition of RSA information for each amino acid compared with the regular (RSA-free) GOR method using leave-one-out cross-validation and different thresholds in two state classification of RSA.

Then we decided to find out whether different amino acids show similar improvement trends. The results for the GOR method are presented in Figure 2. It has not shown a promising picture for the prediction improvement, because the behaviors of some amino acids are opposite. For example, Lys (K) is best predicted with the 16% RSA threshold, while the prediction of Tyr (Y) is the worst by this threshold. In addition, the prediction of some amino acids as Ile (I) always becomes considerably worse with the addition of RSA information, independent of the selected threshold for RSA. The results for Chou-Fasman and HMM methods were generally the same.

While these results prove that the addition of RSA information with a fixed cutoff is not a good recipe for improvement of SS prediction, it clearly shows that one should choose different thresholds for different amino acids (see below).

### Application of residue-specific RSA thresholds for the improvement of secondary structure prediction

In the previous section, we have shown that with the application of a fixed threshold one cannot obtain improvement for all residues. This is something previously observed by Macdonald and Johnson [32], who reported that proline (P) is always considered "buried" in their analysis (they used a fixed threshold of 50% for RSA). Since with the selection of a fixed RSA threshold the predictions of all residues are not improved, we decided to consider "residue-specific" RSA thresholds.

We tested the usefulness of "mean RSA" and "median RSA", i.e. to assume them as the thresholds for each residue *X*. We first obtained the actual distribution of RSA values for each of the twenty amino acids, and then calculated the mean and the median of each of these distributions (see Additional file 6). Then, in two separate tests, the mean and the median were used as residue-specific RSA thresholds.

Table 1 shows the percentage of improvement obtained with the consideration of mean RSA and median RSA as the thresholds for the SS prediction using GOR method. The results are also compared with the fixed 16% threshold, which appeared to be the best cutoffs for the improvement of predictions (Section 3.1.). Obviously, better prediction accuracies are obtained with the consideration of mean RSA and median RSA as the RSA thresholds. However, the amino acids whose predictions are improved are (generally) the same as the amino acids that show prediction improvements with the fixed threshold of 16%. Especially, for Cys, Glu, Ile, Met, Gln, Val and Trp, no improvement is obtained. This means that, the secondary structure propensity for some amino acids is not directly related to their position in surface or core of proteins and two-state surface accessibility classification might not be the best possible way to incorporate RSA information for prediction of secondary structures.

**Table 1:** Improvement of protein secondary structure prediction with the addition of a "residue-specific" RSA threshold using leave-one-out cross-validation, compared with this improvement using a fixed 16% RSA threshold.

	Applied Threshold		
	
	Fixed (16%)	Mean	Median
A	**0.67**	**3.93**	**2.25**
C	-5.02	-0.05	-0.42
D	-1.5	-1.26	-0.94
E	-2.77	**1.33**	-3.71
F	**0.18**	**5.99**	**7.15**
G	**0.90**	**5.98**	**5.53**
H	**0.04**	-4.23	-4.80
I	-19.00	-16.19	-16.17
K	**9.53**	**9.91**	**11.63**
L	**0.54**	**4.20**	**2.24**
M	-6.82	-7.71	-8.29
N	**1.21**	**1.71**	**1.63**
P	**1.15**	**1.70**	**1.47**
Q	-0.61	-1.49	-3.38
R	**1.84**	**0.87**	**1.15**
S	**1.29**	**6.44**	**4.85**
T	**0.20**	**3.12**	**2.53**
V	-2.57	-5.80	-8.33
W	-6.18	-1.66	-2.22
Y	-0.19	**10.04**	**9.78**
			
Total Improvement	**3.46**	**5.79**	**5.13**

The values show percentage of improvement. The bold-underlined values are those values that show improvements when they are compared with the original GOR method. See the text for more details.

We then studied the effect of consideration of three-state residue specific RSA information in SS prediction problem. We tested two types of thresholds again. For the first analysis we chose (mean + SD) and (mean - SD) of the RSA distributions as the selected pair of thresholds. For the second analysis, in case of each amino acid RSA distribution, two RSA values, *t*_1 _and *t*_2 _were selected so that one-third and two-third of the observations were smaller than *t*_1 _and *t*_2_, respectively. We will refer to *t*_1 _and *t*_2 _as the first tertile and the second tertile, respectively. These values are summarized in Additional file 6.

Table 2 shows the percentage of improvement obtained with the consideration of mean RSA and median RSA as the thresholds for the SS prediction compared with [4%, 16%] RSA threshold. While SS prediction shows significant improvements (by more than 7–8%), prediction of the SS of 13 and 15 residues are also improved, while this number had been 11 or 12 in case of two-state RSA classifications. Altogether, all residues except Met and Ile show some level of improvement at least for one of the 6 above classifications (see Tables 1 and 2). This is a very promising result, which suggests that consideration of RSA information can be effectively used for the prediction of SS in proteins. No improvement was obtained in case of Met and Ile, which have highly biased RSA distributions (data not shown). However, there might be some RSA classification assumptions by which SS prediction of these two amino acids are also improved.

**Table 2:** Improvement of protein secondary structure prediction with the addition of two "residue-specific" RSA thresholds, compared with this improvement using a fixed [4%, 16%] RSA threshold.

	Applied Threshold		
	
	Fixed([4%,16%])	Mean ± standard deviation	Tertiles
A	-3.03	-0.83	-0.93
C	**2.63**	**1.92**	**0.74**
D	-0.95	**1.70**	**1.50**
E	-2.54	-1.02	**3.98**
F	**0.71**	**10.14**	**9.44**
G	**0.90**	**9.28**	**7.94**
H	-4.30	-2.14	-3.30
I	-7.49	-14.49	-15.16
K	**10.37**	**26.73**	**13.31**
L	**3.04**	**4.14**	**2.87**
M	-3.58	-5.45	-5.57
N	-1.21	**2.46**	-0.10
P	**1.14**	**2.47**	**1.84**
Q	**0.53**	**0.07**	-0.76
R	**2.80**	**5.10**	**3.04**
S	**4.36**	**13.13**	**12.32**
T	**3.27**	**8.84**	**5.81**
V	**1.57**	**0.13**	-5.72
W	-2.30	**0.40**	**0.25**
Y	**4.17**	**9.73**	**10.30**
			
Total Improvement	**5.44**	**8.24**	**7.17**

The values show percentage of improvement using leave-one-out cross-validation. The bold-underlined values are those values that show improvements when they are compared with the original GOR method. See the text for more details.

In the next step, we tried to see if the effect of adding the RSA information is dependent on the SS prediction method. Table 3 summarizes the results. Clearly, great improvements are also obtained when Chou-Fasman and HMM are used for SS prediction. Interestingly, prediction of the two challenging residues, Met and Ile, shows some improvement here.

**Table 3:** Improvement of protein secondary structure prediction with the addition of a "residue-specific" RSA threshold for Chou-Fasman and HMM method.

	Applied Threshold			
	Chou-Fasman		HMM	
	
	Mean	Median	Mean	Median

A	**11.89**	**10.43**	-5.29	-5.41
C	**3.33**	**1.27**	**4.26**	**4.93**
D	**11.73**	**10.77**	**5.81**	**6.66**
E	**9.16**	**8.56**	-3.55	-3.76
F	-0.32	-0.39	**1.12**	**1.55**
G	**9.11**	**6.72**	**12.79**	**14.25**
H	**10.92**	**12.61**	**2.83**	**3.28**
I	-0.01	-1.45	**0.08**	**0.41**
K	**8.31**	**5.76**	**0.25**	**0.35**
L	**1.08**	**1.21**	-3.53	-3.49
M	**0.17**	-0.40	-3.60	-3.62
N	**8.38**	**8.71**	**7.20**	**8.12**
P	**12.32**	**10.08**	**11.97**	**13.56**
Q	**10.35**	**9.07**	-2.55	-2.53
R	**9.67**	**8.32**	-1.21	-1.10
S	**11.61**	**7.89**	**5.07**	**5.79**
T	**1.68**	**0.16**	**5.22**	**6.10**
V	-0.23	-0.61	**2.20**	**2.50**
W	-0.57	-0.71	-0.78	-0.84
Y	**0.74**	**0.68**	**0.87**	**1.04**
				
Total Improvement	**9.99**	**8.69**	**3.37**	**3.92**

	Applied Threshold			

	Chou-Fasman		HMM	
	
	Tertile	Mean ± standard deviation	Tertile	Mean ± standard deviation

A	**12.15**	**12.50**	-4.31	-2.69
C	**2.64**	**1.76**	**3.85**	**2.93**
D	**13.61**	**13.17**	**8.94**	**6.01**
E	**10.35**	**9.48**	-1.97	-1.04
F	-0.23	-1.88	-0.17	**1.37**
G	**9.29**	**8.80**	**18.48**	**13.14**
H	**12.23**	**11.60**	**4.48**	**3.76**
I	-0.51	**0.09**	-0.41	**0.21**
K	**8.20**	**8.57**	**1.17**	**1.34**
L	**0.72**	**0.49**	-3.35	-1.79
M	**1.76**	-0.81	-1.42	-1.64
N	**8.40**	**8.46**	**10.72**	**7.05**
P	**12.72**	**14.91**	**17.33**	**11.05**
Q	**10.40**	**10.65**	-0.73	-0.66
R	**9.57**	**9.99**	**0.28**	**0.37**
S	**10.10**	**13.09**	**7.41**	**5.28**
T	**0.52**	**0.60**	**6.44**	**5.30**
V	-0.28	-0.20	**0.97**	**2.02**
W	-0.87	-0.63	-0.50	**0.44**
Y	**0.75**	**0.92**	**1.15**	**1.25**
				
Total Improvement	**10.23**	**10.34**	**4.32**	**3.62**

The bold-underlined values are those values that show improvements using leave-one-out cross-validation when they are compared with the original method. See the text for more details.

Our results clearly suggest that considerable improvements are obtained in SS prediction independent of the applied method. It is also important to test the validity of this observation for more popular methods like PSIpred[19] and PHD[18], which work based on finding conserved sequences that form regular structures. However, this is not an easy task. Our approach works by changing the twenty-letter alphabet of amino acids; therefore it is not possible to do the BLAST search with BLOSUM, PAM, or any other classical 20 × 20 matrix, as we need mutation matrices in which RSA information is also considered.

Finally, to assess the usefulness of our suggested residue-specific thresholds, we tried to test the effect of considering random thresholds for classification of RSA data. In each simulation, we randomly assigned one or two thresholds to each amino acid and classified the residues into two or three classes respectively. Then, with the addition of RSA information we computed the prediction accuracy. This procedure was repeated 100 times. The results of the simulation are summarized in Additional file 7. It can be observed that in almost all cases the improvement of the accuracy of prediction is not as high as the suggested residue specific thresholds.

### Application of predicted RSA values for the improvement of secondary structure prediction: can we use real-value RSAs?

We demonstrated that RSA information can positively influence the protein SS prediction. However, in practice, we only know the sequence of the protein, and we may only rely on the predicted RSA values for the improvement, not on the actual values.

Adamczak et al. have previously shown that the predicted real-value RSA information can be used to enhance SS prediction [31]. We used predicted values to test the validity of our approach for this case.

For obtaining predicted RSAs we used RVP-net program [44] to predict RSAs for a given protein sequence in our dataset, and then implemented these predicted RSAs into our method.

**Figure 3:**
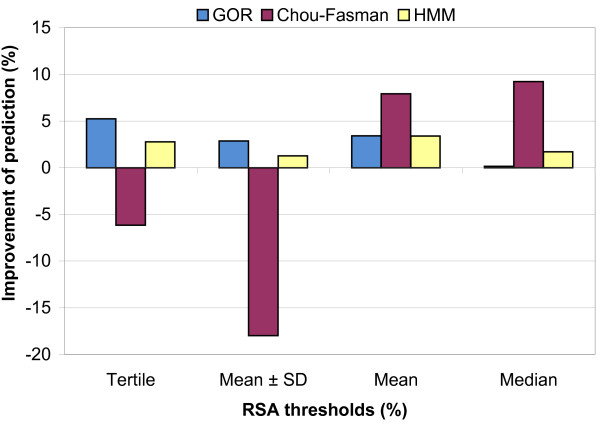
Percentage of improvement in secondary structure prediction accuracy by addition of RSA information for the GOR (A), Chou-Fasman (B) and HMM(C) methods using leave-one-out cross-validation and tertile, Mean ± SD, mean and median as RSA thresholds.

For fixed thresholds, the prediction accuracy dropped by 0.17% to 8.26% (data not shown). When we used means or medians as the residue-specific thresholds, the prediction accuracy was more than original method in all cases. However, when we used tertiles or mean ± standard deviation as the thresholds, the resulting accuracies were more than original method in GOR and HMM methods, but surprisingly, not in Chou-Fasman method (Figure 3).

**Figure 4:**
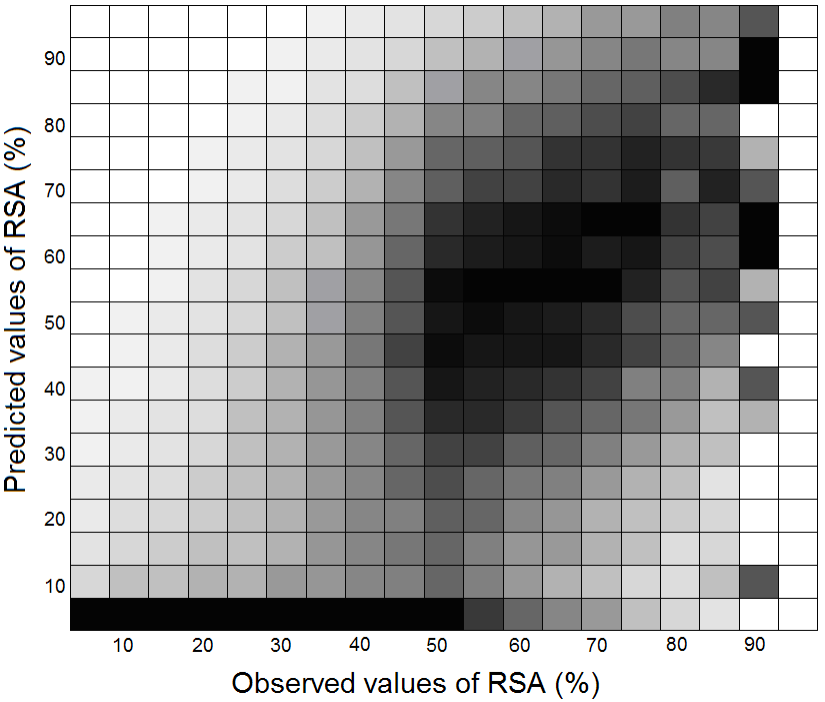
Correlations between observed and predicted values of RSA for different ranges of solvent exposure, scaled to [0,1] interval. The density of vectors is normalized in each column independently. Boxes with maximum density are marked in black, while boxes with minimum density are shown in white. Other colors are selected proportionally to the densities.

The reason for such a difference lies presumably in the nature of Chou-Fasman algorithm. In this algorithm one must first calculate helix and strand residues and then predict the coil residues. The RSA for strand residues are generally less than 50%. We used RVP-net program to predict the required RSAs. Correlations between observed and predicted values of RSA for different ranges of solvent exposure are shown in Figure 4. This Figure suggests that residues with RSA less than 50% are generally significantly underestimated. Thus when we used these data for SS prediction, residues in strand conformation might be inaccurately predicted. In Chou-Fasman algorithm this will also result in incorrect prediction of coils. For two-state RSA assumption, this problem is not a major one, since many residues in each class are still predicted correctly. However, when we classified the RSA data into three groups (using residue specific thresholds, which are typically less than 50%) this problem was intensified, since for the residues with the intermediate RSA, only a small ratio of them are correctly classified as intermediate, and most of them were wrongly categorized as buried.

## Conclusion

In this study we have shown that, combination of actual and predicted RSA greatly improves the prediction of protein secondary structure. In practice, one cannot take advantage of the actual RSA information and it is necessary to use predicted RSA values for this purpose. However, one should notice that RSA prediction methods are still far from being faultless. Therefore, it is critically important to consider the weak points of RSA prediction methods when incorporating their results into SS prediction methods.

## Methods

### Dataset

We used WHATIF [45] PDB selection list, released in January 13, 2007. This dataset contained 6970 chains that have R-factor < 0.25 and resolution < 2.5 Å. The procedure used to generate this dataset was comparable to the PDBselect [46] algorithm, but instead of focusing on maximization of size of the subsets, WHATIF focuses on getting representative structures of the highest available quality. For the WHATIF selection an empirical quality value is defined. This is a composite score depending on the Resolution and the R-factor.

The above dataset was used for training and testing tasks in both the leave-one-out cross-validation and five-fold cross-validation procedure (see below).

### Chou-Fasman method

This method uses a conformational propensity table to predict SS from an input sequence. For each amino acid, this table gives a value describing the given amino acid's propensity to be found in helical structure (H), a strand (E), or in other structures (coil, C). These propensities are calculated by measuring the frequencies of each amino acid associated with a given structure. Then the frequencies were normalized by the prevalence of the amino acid in the dataset.

Using these values, the algorithm looks for "nucleation sites" where either 4 of 6 residues are helix formers or 3 of 5 residues are strand formers. These nucleation sites were then extended as long as the propensity for the given structure remained.

The algorithm also contained additional heuristics for strands, exceptional cases, and others. In this work, these small heuristic amendments are neglected.

In order to add RSA information in this method we classified amino acids into either two or three (i.e. {B(uried), Ex(posed)} or {B(uried), I(ntermediate), Ex(posed)}) discrete groups according to their RSAs. Then, we calculated the propensities of the twenty amino acids, each classified in one of the two or three groups defined based on RSA, and predicted the SS of a given sequence according to this newly built table.

### GOR method

The GOR algorithm [3] and later its newer versions [47], have always been of the most popular methods for SS prediction. The earliest version of GOR had been based on information theory [48], that was introduced by Shannon [49,50] and Fano [51].

In GOR method, for each residue to be predicted, sum of directional information of eight flanking residues on each side is calculated. To obtain the information values from the dataset, the frequency of each of the twenty amino acids at different positions, up to eight residues on the N-terminal and C-terminal sides, should be calculated.

We used GOR IV [13] algorithm, which takes into account another approximation. In this version of GOR, the assumption is made that certain pair-wise combinations of amino acids in the flanking region, influence the conformation of the central amino acid. Hence the information contents calculation formula somewhat changes.

In order to add RSA in these quantities one must further classify residues. This means that instead of 20 residues in three SS conformation, we have 20 residues in 6 combination of SS conformation and RSA states (for two-state classification i.e. {*H*, *E*, *C*} × {*B*(*uried*), *Ex*(*posed*)}). For three-state classification we have 9 combinations of SS conformation and RSA states, i.e. {*H*, *E*, *C*} × {*B*(*uried*), *I*(*ntermediate*), *Ex*(*posed*)}.

### HMM method

In Hidden Markov Models a stochastic model is trained by several sequences, to estimate the probabilities of emissions and transitions. If stochastic models are trained by sequences that have known structures or known functions, the structures and functions for a new sequence can be determined in a stochastic manner, by calculating the probability of the sequence being generated by the model.

Here we first trained three HMMs of Helix, Strand and Coil by training dataset. In order to train the HMMs we calculated the emission probabilities, the transition probabilities and the initial probabilities by measuring the frequencies of amino acids in each structure and each transition. Then we determined the most probable path of a given sequence using Viterbi algorithm[52]. We tested this system by considering the 20 amino acids as the discrete output symbol of HMMs.

In order to implement RSA in this algorithm we divided amino acids into either two or three discrete groups according to their RSAs and trained our models with the resulting either 40 or 60 states.

### RSA and secondary structure assignment

The secondary structure was assigned using DSSP software [41]. In addition, we used the ASA (Accessible Surface Area) from DSSP to determine RSA of each residue by dividing the corresponding ASA value by the maximum possible ASA for each amino acid.

### RSA prediction

We used RVP-net [44] for predicting RSA values. The output of this program is an RSA value between 0% and 100%. We used this value for classifying residues into either two (Buried, Exposed), or three (Buried, Intermediate, Exposed) classes.

### Cross-validation

#### Leave-one-out cross-validation (LOOCV)

This procedure involves removing one chain from the original training set (which contain 6970 chains), using the remaining chains as the training set and then predicting the SS of the removed chain. This process was repeated until all chains have been left out. The final reported values in this work are actually average values over these 6970 experiments.

#### Five-fold cross-validation

We divide randomly the training set into 5 parts, four of which are used for training and the rest for testing. This process is repeated 10 times to ensure that the order of the chains that are used, do not affect the prediction.

### Accuracy measures for evaluation of prediction

**Q_3_: **Prediction accuracy has been assessed by the percentage of correctly predicted residues (Q_3_) for a three-state description of secondary structure (Helix, Strand and Coil), where Q_3 _is the percentage of amino acids correctly predicted as helix, sheet, or coil if all amino acids are classified in one of the three groups.

The value of Q_3 _is calculated using the following formula:

(1) $$Q_{3}=\frac{\sum_{\text{X}=\text{H,S,C}}\text{Number of correctly predicted amino acids in structure X}}{\text{Total number of amino acids}}\times 100$$

#### Standard deviation

The standard deviation is defined by:

(2) $$SD=\sqrt{\frac{\sum (X_{i}-\bar{X}{)}^{2}}{n-1}}$$

where *X*_*i *_is our variable, $\bar{X}$ is the mean and n is the total number of observations. In this study we calculate two different standard deviations. The first one that is used in LOOCV is the standard deviation of Q_3 _of 6961 chains and the second one which is used in Five-fold cross-validation is the standard deviation of Q_3 _in 10-time repeated cross-validation.

## Authors' contributions

All authors participated in the design of the study. AMR implemented the method. SAM, AMR and MS were involved in interpreting the results. The original manuscript was drafted by SAM and completed by AMR, MS and HP. All authors read and approved the final manuscript.

## Supplementary Material

Additional file 1 Accuracy of secondary structure prediction for GOR, Chou-Fasman and HMM methods, without consideration of RSA information. Click here for file

Additional file 2 Accuracy of secondary structure prediction for GOR method, with the consideration of actual and predicted RSA information. Click here for file

Additional file 3 Accuracy of secondary structure prediction for Chou-Fasman method, with the consideration of actual and predicted RSA information. Click here for file

Additional file 4 Accuracy of secondary structure prediction for HMM method, with the consideration of actual and predicted RSA information. Click here for file

Additional file 5 Percentage of improvement in secondary structure prediction accuracy compared with the GOR (A), Chou-Fasman (B) and HMM(C) methods using different thresholds in three-state classification of RSA. Click here for file

Additional file 6 Applied residue-specific thresholds used for classification of RSA values. Click here for file

Additional file 7 Accuracy of secondary structure prediction for GOR, Chou-Fasman and HMM methods, with the consideration of random two- and three-state classification of actual RSA information. Click here for file
