# Supplementary material for: Impact of residue accessible surface area on the prediction of protein secondary structures
Source: BMC Bioinformatics. 2008 Aug 31;9:357. doi: 10.1186/1471-2105-9-357 (PMC2553345; doi:10.1186/1471-2105-9-357)
Supplement: Additional file 1 — Accuracy of secondary structure prediction for GOR, Chou-Fasman and HMM methods, without consideration of RSA information. [file 1471-2105-9-357-S1.doc]

A) Accuracy of secondary structure prediction for GOR, Chou-Fasman and HMM methods using leave-one-out cross-validation, without consideration of RSA information. Totally, 1571044 residues were present in the assessed dataset. For each of the twenty amino acids the accuracy of prediction is reported separately.

|  |  | Method | | |
| --- | --- | --- | --- | --- |
|  |  | GOR | Chou-Fasman | HMM |
|  |  |  |  |  |
| Correct |  | 786860 | 693537 | 713806 |
| False |  | 784184 | 877507 | 857238 |
| Q3 |  | 49.732 | 44.145 | 45.966 |
| SD |  | 11.430 | 10.643 | 16.617 |
|  |  |  |  |  |
| A |  | 56.762 | 47.775 | 52.925 |
| C |  | 48.095 | 43.822 | 41.549 |
| D |  | 54.458 | 39.764 | 45.288 |
| E |  | 54.585 | 47.906 | 52.552 |
| F |  | 41.109 | 44.208 | 41.444 |
| G |  | 66.583 | 38.141 | 42.777 |
| H |  | 51.787 | 42.313 | 44.019 |
| I |  | 58.715 | 48.621 | 40.005 |
| K |  | 51.939 | 43.256 | 48.009 |
| L |  | 51.349 | 47.400 | 47.844 |
| M |  | 55.481 | 44.598 | 48.913 |
| N |  | 57.094 | 38.924 | 45.093 |
| P |  | 69.090 | 41.535 | 46.344 |
| Q |  | 55.959 | 44.694 | 50.596 |
| R |  | 53.395 | 42.958 | 47.868 |
| S |  | 49.410 | 40.864 | 43.823 |
| T |  | 46.036 | 41.852 | 40.871 |
| V |  | 55.582 | 50.035 | 37.136 |
| W |  | 48.463 | 43.133 | 43.124 |
| Y |  | 40.371 | 43.296 | 41.559 |
|  |  |  |  |  |

B) Accuracy of secondary structure prediction for GOR, Chou-Fasman and HMM methods using Five-fold cross-validation, without consideration of RSA information

|  |  | Method | | | | | |
| --- | --- | --- | --- | --- | --- | --- | --- |
|  |  | GOR | | Chou-Fasman | | HMM | |
|  |  |  | |  | |  | |
| Correct |  | 792200.194 | | 730747.542 | | 697000.627 | |
| False |  | 778843.806 | | 840296.458 | | 874043.373 | |
|  |  | Q3 | SD | Q3 | SD | Q3 | SD |
| Total |  | 50.425 | 1.085 | 46.513 | 0.036 | 44.365 | 1.312 |
|  |  |  |  |  |  |  |  |
| A |  | 56.639 | 0.087 | 27.031 | 0.790 | 52.586 | 1.459 |
| C |  | 47.813 | 0.275 | 22.363 | 0.410 | 40.226 | 0.709 |
| D |  | 54.461 | 1.571 | 34.741 | 0.447 | 43.765 | 1.350 |
| E |  | 54.312 | 0.114 | 18.844 | 0.516 | 52.190 | 1.315 |
| F |  | 41.338 | 0.814 | 20.812 | 0.447 | 40.784 | 1.314 |
| G |  | 66.585 | 2.240 | 33.919 | 0.706 | 39.968 | 1.356 |
| H |  | 50.968 | 0.625 | 22.706 | 0.234 | 42.903 | 0.853 |
| I |  | 57.843 | 0.565 | 27.029 | 0.623 | 39.530 | 2.117 |
| K |  | 52.713 | 1.576 | 40.057 | 0.328 | 47.276 | 1.301 |
| L |  | 51.081 | 0.124 | 22.551 | 0.822 | 47.607 | 0.778 |
| M |  | 55.254 | 0.166 | 28.698 | 0.884 | 48.226 | 1.612 |
| N |  | 57.097 | 0.958 | 26.963 | 0.337 | 43.126 | 1.679 |
| P |  | 69.090 | 0.749 | 20.456 | 0.855 | 43.474 | 1.114 |
| Q |  | 55.923 | 0.718 | 19.242 | 0.610 | 50.017 | 1.162 |
| R |  | 53.701 | 0.750 | 22.336 | 0.465 | 47.269 | 0.948 |
| S |  | 49.604 | 1.220 | 24.428 | 0.378 | 42.277 | 1.089 |
| T |  | 45.992 | 0.677 | 24.666 | 0.622 | 39.384 | 1.189 |
| V |  | 54.695 | 1.070 | 30.590 | 0.573 | 36.391 | 1.339 |
| W |  | 48.627 | 0.063 | 43.885 | 0.280 | 42.574 | 1.069 |
| Y |  | 40.533 | 0.629 | 31.185 | 0.209 | 40.789 | 0.131 |
|  |  |  | |  | |  | |
