# Supplementary material for: Impact of residue accessible surface area on the prediction of protein secondary structures
Source: BMC Bioinformatics. 2008 Aug 31;9:357. doi: 10.1186/1471-2105-9-357 (PMC2553345; doi:10.1186/1471-2105-9-357)
Supplement: Additional file 2 — Accuracy of secondary structure prediction for GOR method, with the consideration of actual and predicted RSA information. [file 1471-2105-9-357-S2.doc]

A) Accuracy of secondary structure prediction for GOR method using leave-one-out cross-validation, with the consideration of actual two-state RSA information. Totally, 1571044 residues were present in the assessed dataset. For each of the twenty amino acids the accuracy of prediction is reported separately.

|  |  | Thresholds | | | | | |
| --- | --- | --- | --- | --- | --- | --- | --- |
|  |  | 4 | 9 | 16 | 25 | 36 | 50 |
| Correct |  | 824040 | 824214 | 841259 | 832444 | 826146 | 816342 |
| False |  | 747004 | 746830 | 729785 | 738600 | 744898 | 754702 |
| Q3 |  | 52.243 | 52.278 | 53.184 | 52.710 | 52.352 | 51.790 |
| SD |  | 10.057 | 10.137 | 9.534 | 9.736 | 9.945 | 10.209 |
|  |  |  |  |  |  |  |  |
| A |  | 56.516 | 52.671 | 57.432 | 56.505 | 56.461 | 53.460 |
| C |  | 40.676 | 40.639 | 43.074 | 40.986 | 40.690 | 40.658 |
| D |  | 55.396 | 55.396 | 55.396 | 55.396 | 55.396 | 55.396 |
| E |  | 53.763 | 53.676 | 51.813 | 52.155 | 53.103 | 57.774 |
| F |  | 45.691 | 44.120 | 41.293 | 43.070 | 45.007 | 46.917 |
| G |  | 67.487 | 67.487 | 67.487 | 67.487 | 67.487 | 67.487 |
| H |  | 44.885 | 43.604 | 51.832 | 49.025 | 45.768 | 44.404 |
| I |  | 38.878 | 37.610 | 39.716 | 38.955 | 38.889 | 38.898 |
| K |  | 51.416 | 56.316 | 61.469 | 59.005 | 53.470 | 48.702 |
| L |  | 48.580 | 47.723 | 51.890 | 49.627 | 48.530 | 48.511 |
| M |  | 48.563 | 45.427 | 48.664 | 48.695 | 48.688 | 48.390 |
| N |  | 58.301 | 58.301 | 58.301 | 58.301 | 58.301 | 58.301 |
| P |  | 70.239 | 70.239 | 70.239 | 70.239 | 70.239 | 70.239 |
| Q |  | 57.838 | 48.854 | 55.346 | 56.421 | 57.601 | 54.661 |
| R |  | 54.035 | 50.399 | 55.235 | 55.808 | 55.225 | 49.495 |
| S |  | 50.701 | 50.701 | 50.701 | 50.701 | 50.701 | 50.701 |
| T |  | 45.072 | 45.072 | 46.239 | 45.093 | 45.072 | 45.072 |
| V |  | 47.782 | 58.454 | 53.015 | 50.657 | 48.528 | 46.133 |
| W |  | 43.055 | 40.229 | 42.278 | 42.310 | 42.802 | 43.574 |
| Y |  | 46.835 | 50.712 | 40.185 | 42.597 | 45.889 | 49.043 |
|  |  |  |  |  |  |  |  |

B) Accuracy of secondary structure prediction for GOR method using leave-one-out cross-validation, with the consideration of predicted two-state RSA information.

|  |  | Thresholds | | | | | |
| --- | --- | --- | --- | --- | --- | --- | --- |
|  |  | 4 | 9 | 16 | 25 | 36 | 50 |
| Correct |  | 765293 | 702607 | 811136 | 804362 | 794147 | 787818 |
| False |  | 805751 | 868437 | 759908 | 766682 | 776897 | 783226 |
| Q3 |  | 48.167 | 43.983 | 51.289 | 50.831 | 50.167 | 49.781 |
| SD |  | 7.488 | 8.844 | 10.524 | 10.644 | 10.460 | 11.009 |
|  |  |  |  |  |  |  |  |
| A |  | 52.301 | 38.754 | 54.387 | 52.794 | 52.163 | 51.986 |
| C |  | 40.639 | 36.062 | 42.732 | 40.870 | 40.662 | 40.644 |
| D |  | 55.396 | 55.396 | 55.396 | 55.396 | 55.396 | 55.396 |
| E |  | 54.451 | 33.408 | 51.589 | 51.941 | 52.806 | 55.498 |
| F |  | 35.343 | 32.001 | 39.576 | 40.697 | 41.966 | 42.707 |
| G |  | 67.487 | 67.487 | 67.487 | 67.487 | 67.487 | 67.487 |
| H |  | 43.604 | 43.604 | 45.568 | 47.605 | 45.186 | 44.155 |
| I |  | 37.759 | 33.258 | 37.556 | 37.443 | 37.363 | 37.313 |
| K |  | 42.188 | 39.824 | 58.643 | 55.206 | 47.588 | 46.426 |
| L |  | 40.792 | 42.850 | 47.752 | 47.644 | 47.642 | 47.645 |
| M |  | 48.733 | 42.190 | 44.519 | 44.460 | 44.488 | 44.508 |
| N |  | 58.301 | 58.301 | 58.301 | 58.301 | 58.301 | 58.301 |
| P |  | 70.239 | 70.239 | 70.239 | 70.239 | 70.239 | 70.239 |
| Q |  | 57.282 | 36.057 | 53.405 | 54.735 | 54.953 | 50.734 |
| R |  | 50.718 | 36.389 | 52.817 | 53.316 | 51.236 | 46.791 |
| S |  | 50.701 | 50.701 | 50.701 | 50.701 | 50.701 | 50.701 |
| T |  | 45.072 | 45.072 | 45.372 | 45.077 | 45.072 | 45.072 |
| V |  | 35.830 | 43.286 | 46.889 | 44.026 | 42.014 | 38.790 |
| W |  | 33.006 | 36.089 | 42.168 | 40.532 | 40.601 | 41.148 |
| Y |  | 33.229 | 33.475 | 40.871 | 40.651 | 42.116 | 44.958 |
|  |  |  |  |  |  |  |  |

C) Accuracy of secondary structure prediction for GOR method using leave-one-out cross-validation, with the consideration of actual three-state RSA information.

|  |  | Thresholds | | | |
| --- | --- | --- | --- | --- | --- |
|  |  | [4,16] | [9,16] | [9,36] | [16,36] |
| Correct |  | 875083 | 861481 | 862959 | 846468 |
| False |  | 695961 | 709563 | 708085 | 724576 |
| Q3 |  | 55.170 | 54.384 | 54.550 | 53.573 |
| SD |  | 9.334 | 9.441 | 9.354 | 9.445 |
|  |  |  |  |  |  |
| A |  | 59.790 | 59.480 | 60.037 | 58.374 |
| C |  | 50.455 | 51.933 | 52.390 | 44.062 |
| D |  | 55.408 | 55.396 | 55.396 | 55.396 |
| E |  | 52.049 | 51.934 | 54.129 | 53.841 |
| F |  | 41.816 | 41.226 | 41.307 | 41.836 |
| G |  | 67.487 | 67.487 | 67.487 | 67.487 |
| H |  | 47.488 | 50.463 | 50.414 | 52.188 |
| I |  | 51.223 | 44.091 | 46.528 | 42.335 |
| K |  | 62.306 | 61.799 | 55.815 | 55.505 |
| L |  | 54.389 | 52.817 | 50.843 | 50.158 |
| M |  | 51.898 | 50.348 | 54.157 | 52.986 |
| N |  | 58.304 | 58.301 | 58.301 | 58.301 |
| P |  | 70.239 | 70.239 | 70.239 | 70.239 |
| Q |  | 56.486 | 56.089 | 58.603 | 58.490 |
| R |  | 56.194 | 55.938 | 56.218 | 56.030 |
| S |  | 53.772 | 50.872 | 50.893 | 50.701 |
| T |  | 49.308 | 50.672 | 50.694 | 46.483 |
| V |  | 57.145 | 56.028 | 56.541 | 54.319 |
| W |  | 46.161 | 44.714 | 48.794 | 45.247 |
| Y |  | 44.537 | 42.411 | 42.687 | 41.470 |
|  |  |  |  |  |  |

D) Accuracy of secondary structure prediction for GOR method using leave-one-out cross-validation, with the consideration of predicted three-state RSA information.

|  |  | Thresholds | | | |
| --- | --- | --- | --- | --- | --- |
|  |  | [4,16] | [9,16] | [9,36] | [16,36] |
| Correct |  | 820652 | 827356 | 818520 | 803845 |
| False |  | 750392 | 743688 | 752524 | 767199 |
| Q3 |  | 51.797 | 52.299 | 51.730 | 50.810 |
| SD |  | 9.461 | 9.795 | 9.332 | 10.125 |
|  |  |  |  |  |  |
| A |  | 57.123 | 57.744 | 56.791 | 53.706 |
| C |  | 45.235 | 48.173 | 48.506 | 43.189 |
| D |  | 55.396 | 55.396 | 55.396 | 55.396 |
| E |  | 51.537 | 51.638 | 53.057 | 52.767 |
| F |  | 35.272 | 40.930 | 40.888 | 39.414 |
| G |  | 67.487 | 67.487 | 67.487 | 67.487 |
| H |  | 43.637 | 43.672 | 43.639 | 45.641 |
| I |  | 45.155 | 39.239 | 39.857 | 37.693 |
| K |  | 58.273 | 58.206 | 49.147 | 49.904 |
| L |  | 50.913 | 48.202 | 48.227 | 47.705 |
| M |  | 47.749 | 45.053 | 45.631 | 44.512 |
| N |  | 58.301 | 58.301 | 58.301 | 58.301 |
| P |  | 70.239 | 70.239 | 70.239 | 70.239 |
| Q |  | 53.002 | 53.645 | 54.569 | 54.515 |
| R |  | 53.104 | 53.425 | 51.353 | 51.128 |
| S |  | 50.701 | 50.702 | 50.703 | 50.701 |
| T |  | 45.072 | 45.152 | 45.148 | 45.441 |
| V |  | 40.907 | 51.905 | 52.647 | 47.631 |
| W |  | 46.239 | 44.318 | 46.933 | 42.306 |
| Y |  | 46.662 | 44.591 | 41.152 | 40.961 |
|  |  |  |  |  |  |

E) Accuracy of secondary structure prediction for GOR method using leave-one-out cross-validation, with the consideration of residue-specific classification of actual RSA information.

|  |  | Thresholds | | | |
| --- | --- | --- | --- | --- | --- |
|  |  | Tertiles | Mean±SD | Mean | Median |
| Correct |  | 901525 | 917549 | 875548 | 856958 |
| False |  | 669519 | 653495 | 695496 | 714086 |
| Q3 |  | 57.384 | 58.404 | 55.730 | 54.547 |
| SD |  | 9.620 | 8.689 | 10.716 | 10.751 |
|  |  |  |  |  |  |
| A |  | 55.829 | 55.936 | 60.693 | 59.015 |
| C |  | 48.830 | 50.013 | 48.049 | 47.679 |
| D |  | 55.962 | 56.162 | 55.717 | 58.418 |
| E |  | 58.570 | 53.563 | 53.257 | 50.877 |
| F |  | 50.545 | 51.250 | 47.095 | 48.257 |
| G |  | 74.524 | 75.863 | 72.560 | 72.115 |
| H |  | 48.485 | 49.650 | 47.552 | 46.984 |
| I |  | 43.551 | 44.226 | 42.529 | 42.543 |
| K |  | 65.252 | 78.669 | 61.846 | 63.565 |
| L |  | 54.214 | 55.488 | 55.544 | 53.585 |
| M |  | 49.908 | 50.031 | 47.775 | 47.191 |
| N |  | 59.435 | 59.552 | 58.802 | 58.721 |
| P |  | 70.931 | 71.556 | 70.793 | 70.560 |
| Q |  | 55.203 | 56.033 | 54.470 | 52.583 |
| R |  | 56.432 | 58.492 | 52.527 | 54.544 |
| S |  | 61.731 | 62.545 | 55.855 | 54.257 |
| T |  | 51.849 | 54.879 | 49.155 | 48.566 |
| V |  | 49.866 | 55.709 | 49.777 | 47.254 |
| W |  | 47.210 | 48.863 | 46.798 | 46.239 |
| Y |  | 50.671 | 50.104 | 50.416 | 50.150 |
|  |  |  |  |  |  |

F) Accuracy of secondary structure prediction for GOR method using leave-one-out cross-validation, with the consideration of residue-specific classification of predicted RSA information.

|  |  | Thresholds | | | |
| --- | --- | --- | --- | --- | --- |
|  |  | Tertiles | Mean±SD | Mean | Median |
| Correct |  | 868317 | 828437 | 839148 | 788566 |
| False |  | 702727 | 742607 | 731896 | 782478 |
| Q3 |  | 54.978 | 52.596 | 53.153 | 49.873 |
| SD |  | 9.290 | 9.681 | 9.343 | 11.405 |
|  |  |  |  |  |  |
| A |  | 57.550 | 56.409 | 55.765 | 51.981 |
| C |  | 52.778 | 52.095 | 48.067 | 42.062 |
| D |  | 54.458 | 54.458 | 54.458 | 54.458 |
| E |  | 60.322 | 54.129 | 53.966 | 51.326 |
| F |  | 48.077 | 40.843 | 40.507 | 40.315 |
| G |  | 66.583 | 66.583 | 66.583 | 66.583 |
| H |  | 48.161 | 45.214 | 52.580 | 42.243 |
| I |  | 56.229 | 40.389 | 48.625 | 37.293 |
| K |  | 52.802 | 53.999 | 51.703 | 55.904 |
| L |  | 51.487 | 50.545 | 50.936 | 47.638 |
| M |  | 50.792 | 48.605 | 49.610 | 44.557 |
| N |  | 57.094 | 57.094 | 57.094 | 57.094 |
| P |  | 69.090 | 69.090 | 69.090 | 69.090 |
| Q |  | 57.158 | 50.159 | 55.855 | 47.812 |
| R |  | 53.696 | 47.919 | 53.306 | 46.522 |
| S |  | 51.679 | 51.744 | 49.410 | 49.410 |
| T |  | 49.305 | 46.282 | 44.073 | 43.512 |
| V |  | 55.483 | 58.421 | 58.677 | 45.108 |
| W |  | 47.121 | 41.998 | 45.881 | 40.339 |
| Y |  | 44.486 | 41.371 | 39.226 | 44.064 |
|  |  |  |  |  |  |

G) Accuracy of secondary structure prediction for GOR method using Five-fold cross-validation, with the consideration of actual two-state RSA information.

|  |  | Thresholds | | | | | |
| --- | --- | --- | --- | --- | --- | --- | --- |
|  |  | 4 | 9 | 16 | 25 | 36 | 50 |
| Correct |  | 832463.901 | 836673.382 | 850950.799 | 842520.545 | 836287.233 | 826793.485 |
| False |  | 738580.099 | 734370.618 | 720093.201 | 728523.455 | 734756.767 | 744250.515 |
| Q3 |  | 52.988 | 53.256 | 54.165 | 53.628 | 53.231 | 52.627 |
|  |  |  |  |  |  |  |  |
| A |  | 57.090 | 54.162 | 58.289 | 57.333 | 57.127 | 54.134 |
| C |  | 41.742 | 41.851 | 43.526 | 41.339 | 40.969 | 40.897 |
| D |  | 55.562 | 55.892 | 55.885 | 55.896 | 55.896 | 55.896 |
| E |  | 53.886 | 54.127 | 52.510 | 52.900 | 53.937 | 58.479 |
| F |  | 45.889 | 44.335 | 41.935 | 43.803 | 45.777 | 47.688 |
| G |  | 67.718 | 68.110 | 68.118 | 68.115 | 68.121 | 68.123 |
| H |  | 45.270 | 44.503 | 51.679 | 48.920 | 45.900 | 44.657 |
| I |  | 40.272 | 39.102 | 40.486 | 39.586 | 39.453 | 39.416 |
| K |  | 52.573 | 57.212 | 61.653 | 59.179 | 53.833 | 49.492 |
| L |  | 49.457 | 48.882 | 52.290 | 50.060 | 49.043 | 49.047 |
| M |  | 48.739 | 46.157 | 49.104 | 49.198 | 49.255 | 49.009 |
| N |  | 58.462 | 58.800 | 58.795 | 58.801 | 58.788 | 58.793 |
| P |  | 70.431 | 70.874 | 70.871 | 70.869 | 70.869 | 70.872 |
| Q |  | 58.124 | 50.595 | 56.432 | 57.406 | 58.300 | 55.041 |
| R |  | 54.727 | 51.861 | 56.180 | 56.628 | 55.808 | 50.193 |
| S |  | 50.927 | 51.128 | 51.108 | 51.108 | 51.110 | 51.106 |
| T |  | 45.664 | 45.753 | 46.532 | 45.421 | 45.396 | 45.394 |
| V |  | 48.953 | 58.885 | 54.235 | 52.221 | 50.317 | 48.126 |
| W |  | 43.504 | 40.836 | 42.786 | 43.018 | 43.698 | 44.592 |
| Y |  | 46.484 | 49.890 | 40.735 | 43.257 | 46.534 | 49.492 |
|  |  |  |  |  |  |  |  |

H) Accuracy of secondary structure prediction for GOR method using Five-fold cross-validation, with the consideration of predicted two-state RSA information.

|  |  | Thresholds | | | | | |
| --- | --- | --- | --- | --- | --- | --- | --- |
|  |  | 4 | 9 | 16 | 25 | 36 | 50 |
| Correct |  | 775564.482 | 716921.334 | 813427.237 | 807273.535 | 797807.867 | 790556.769 |
| False |  | 795479.518 | 854122.666 | 757616.763 | 763770.465 | 773236.133 | 780487.231 |
| Q3 |  | 49.366 | 45.633 | 51.776 | 51.385 | 50.782 | 50.320 |
|  |  |  |  |  |  |  |  |
| A |  | 52.960 | 40.646 | 54.549 | 53.141 | 52.543 | 51.937 |
| C |  | 41.260 | 37.192 | 42.910 | 41.037 | 40.771 | 40.699 |
| D |  | 55.264 | 55.026 | 55.032 | 55.037 | 55.040 | 55.040 |
| E |  | 54.570 | 35.582 | 51.815 | 52.226 | 53.223 | 55.884 |
| F |  | 36.786 | 33.901 | 40.677 | 41.773 | 42.972 | 43.580 |
| G |  | 67.122 | 66.734 | 66.758 | 66.766 | 66.773 | 66.768 |
| H |  | 44.063 | 43.899 | 45.435 | 47.069 | 44.865 | 43.933 |
| I |  | 39.146 | 35.313 | 39.227 | 39.147 | 39.063 | 39.010 |
| K |  | 43.830 | 41.656 | 58.335 | 55.115 | 48.025 | 46.601 |
| L |  | 42.151 | 43.836 | 48.122 | 47.943 | 47.913 | 47.920 |
| M |  | 49.023 | 43.037 | 45.085 | 45.043 | 45.079 | 45.147 |
| N |  | 58.007 | 57.750 | 57.759 | 57.753 | 57.765 | 57.772 |
| P |  | 69.741 | 69.303 | 69.308 | 69.303 | 69.319 | 69.311 |
| Q |  | 57.408 | 38.300 | 53.797 | 54.996 | 55.090 | 50.626 |
| R |  | 51.139 | 38.153 | 52.848 | 53.283 | 51.227 | 46.650 |
| S |  | 50.573 | 50.331 | 50.315 | 50.310 | 50.319 | 50.320 |
| T |  | 45.572 | 45.505 | 45.578 | 45.237 | 45.206 | 45.173 |
| V |  | 38.021 | 44.352 | 47.627 | 45.103 | 43.350 | 40.500 |
| W |  | 34.569 | 37.363 | 42.839 | 41.431 | 41.580 | 42.069 |
| Y |  | 34.631 | 34.913 | 41.555 | 41.459 | 42.788 | 45.196 |
|  |  |  |  |  |  |  |  |

I) Accuracy of secondary structure prediction for GOR method using Five-fold cross-validation, with the consideration of actual three-state RSA information.

|  |  | Thresholds | | | |
| --- | --- | --- | --- | --- | --- |
|  |  | [4,16] | [9,16] | [9,36] | [16,36] |
| Correct |  | 882451.485 | 870596.209 | 871554.889 | 856029.637 |
| False |  | 688592.515 | 700447.791 | 699489.111 | 715014.363 |
| Q3 |  | 56.170 | 55.415 | 55.476 | 54.488 |
|  |  |  |  |  |  |
| A |  | 60.399 | 60.237 | 60.765 | 59.173 |
| C |  | 50.719 | 51.996 | 52.321 | 44.481 |
| D |  | 55.907 | 55.884 | 55.886 | 55.892 |
| E |  | 53.008 | 52.689 | 54.935 | 54.774 |
| F |  | 42.592 | 41.782 | 41.869 | 42.469 |
| G |  | 68.117 | 68.112 | 68.112 | 68.111 |
| H |  | 47.752 | 50.631 | 50.606 | 52.021 |
| I |  | 51.568 | 45.151 | 47.308 | 43.142 |
| K |  | 62.240 | 61.988 | 56.263 | 55.802 |
| L |  | 54.698 | 53.289 | 51.487 | 50.790 |
| M |  | 52.223 | 50.758 | 54.276 | 53.179 |
| N |  | 58.791 | 58.794 | 58.792 | 58.798 |
| P |  | 70.867 | 70.869 | 70.879 | 70.865 |
| Q |  | 57.282 | 57.194 | 59.330 | 59.197 |
| R |  | 56.816 | 56.879 | 56.871 | 56.608 |
| S |  | 53.905 | 51.288 | 51.285 | 51.116 |
| T |  | 49.560 | 50.753 | 50.720 | 46.763 |
| V |  | 57.599 | 56.758 | 57.318 | 55.466 |
| W |  | 46.634 | 45.088 | 48.741 | 45.582 |
| Y |  | 44.934 | 42.664 | 42.873 | 41.941 |
|  |  |  |  |  |  |

J) Accuracy of secondary structure prediction for GOR method using Five-fold cross-validation, with the consideration of predicted three-state RSA information.

|  |  | Thresholds | | | |
| --- | --- | --- | --- | --- | --- |
|  |  | [4,16] | [9,16] | [9,36] | [16,36] |
| Correct |  | 826840.656 | 832107.416 | 823917.430 | 812127.217 |
| False |  | 744203.344 | 738936.584 | 747126.570 | 758916.783 |
| Q3 |  | 52.630 | 52.965 | 52.444 | 51.693 |
|  |  |  |  |  |  |
| A |  | 57.275 | 57.896 | 57.141 | 54.544 |
| C |  | 45.762 | 48.197 | 48.413 | 43.391 |
| D |  | 55.405 | 55.390 | 55.390 | 55.513 |
| E |  | 52.138 | 51.928 | 53.494 | 53.426 |
| F |  | 36.416 | 41.071 | 41.067 | 40.008 |
| G |  | 67.436 | 67.506 | 67.506 | 67.658 |
| H |  | 44.172 | 44.252 | 44.234 | 45.896 |
| I |  | 46.424 | 40.631 | 40.977 | 38.694 |
| K |  | 58.046 | 58.197 | 49.789 | 50.437 |
| L |  | 51.072 | 48.588 | 48.559 | 48.203 |
| M |  | 48.250 | 45.724 | 46.252 | 45.272 |
| N |  | 58.265 | 58.270 | 58.270 | 58.399 |
| P |  | 70.130 | 70.236 | 70.236 | 70.393 |
| Q |  | 53.525 | 54.342 | 55.123 | 55.229 |
| R |  | 53.355 | 53.961 | 51.978 | 51.851 |
| S |  | 50.746 | 50.689 | 50.659 | 50.767 |
| T |  | 45.570 | 45.471 | 45.390 | 45.534 |
| V |  | 42.811 | 52.736 | 53.438 | 49.119 |
| W |  | 46.326 | 44.429 | 46.736 | 42.654 |
| Y |  | 46.501 | 44.335 | 41.225 | 41.220 |
|  |  |  |  |  |  |

K) Accuracy of secondary structure prediction for GOR method using Five-fold cross-validation, with the consideration of residue-specific classification of actual RSA information.

|  |  | Thresholds | | | |
| --- | --- | --- | --- | --- | --- |
|  |  | Tertiles | Mean±SD | Mean | Median |
| Correct |  | 904696.396 | 917795.510 | 884461.670 | 875712.563 |
| False |  | 666347.604 | 653248.490 | 686582.330 | 695331.437 |
| Q3 |  | 57.586 | 58.419 | 56.298 | 55.741 |
|  |  |  |  |  |  |
| A |  | 57.103 | 56.549 | 61.283 | 59.909 |
| C |  | 49.355 | 49.299 | 48.570 | 48.481 |
| D |  | 56.392 | 56.570 | 56.170 | 58.579 |
| E |  | 59.096 | 54.236 | 54.520 | 52.471 |
| F |  | 50.639 | 51.250 | 47.186 | 48.031 |
| G |  | 74.384 | 75.575 | 72.634 | 72.238 |
| H |  | 48.893 | 49.846 | 47.731 | 47.221 |
| I |  | 44.775 | 44.398 | 43.498 | 44.202 |
| K |  | 64.788 | 77.184 | 61.317 | 62.774 |
| L |  | 54.559 | 55.279 | 55.775 | 54.322 |
| M |  | 50.647 | 50.482 | 48.480 | 47.728 |
| N |  | 59.806 | 59.905 | 59.241 | 59.172 |
| P |  | 71.488 | 72.043 | 71.358 | 71.163 |
| Q |  | 55.755 | 56.219 | 55.386 | 53.811 |
| R |  | 56.707 | 58.295 | 53.399 | 55.310 |
| S |  | 61.019 | 61.960 | 55.718 | 54.294 |
| T |  | 51.861 | 54.625 | 49.186 | 48.557 |
| V |  | 51.146 | 56.899 | 51.293 | 48.791 |
| W |  | 47.384 | 49.007 | 46.811 | 46.163 |
| Y |  | 50.337 | 50.380 | 49.968 | 49.554 |
|  |  |  |  |  |  |

L) Accuracy of secondary structure prediction for GOR method using Five-fold cross-validation, with the consideration of residue-specific classification of predicted RSA information.

|  |  | Thresholds | | | |
| --- | --- | --- | --- | --- | --- |
|  |  | Tertiles | Mean±SD | Mean | Median |
| Correct |  | 871398.507 | 826996.864 | 843761.518 | 800558.848 |
| False |  | 699645.493 | 744047.136 | 727282.482 | 770485.152 |
| Q3 |  | 55.466 | 52.640 | 53.707 | 50.957 |
|  |  |  |  |  |  |
| A |  | 58.636 | 55.540 | 56.881 | 53.630 |
| C |  | 53.336 | 51.891 | 48.249 | 43.075 |
| D |  | 55.258 | 55.063 | 55.050 | 55.047 |
| E |  | 60.061 | 53.042 | 54.953 | 52.861 |
| F |  | 47.768 | 41.087 | 40.727 | 40.504 |
| G |  | 67.551 | 67.333 | 67.303 | 67.308 |
| H |  | 48.377 | 45.616 | 52.052 | 42.823 |
| I |  | 55.408 | 41.022 | 47.804 | 37.973 |
| K |  | 52.207 | 53.674 | 51.149 | 54.950 |
| L |  | 52.031 | 49.684 | 51.412 | 48.920 |
| M |  | 50.417 | 48.420 | 49.201 | 44.749 |
| N |  | 57.923 | 57.720 | 57.715 | 57.724 |
| P |  | 70.101 | 69.846 | 69.855 | 69.855 |
| Q |  | 56.803 | 49.858 | 56.365 | 49.600 |
| R |  | 53.426 | 47.806 | 53.692 | 48.142 |
| S |  | 52.174 | 52.205 | 49.985 | 49.986 |
| T |  | 49.453 | 46.778 | 44.576 | 44.048 |
| V |  | 55.060 | 57.196 | 57.832 | 45.777 |
| W |  | 46.370 | 42.170 | 45.121 | 40.208 |
| Y |  | 44.249 | 41.354 | 39.280 | 43.576 |
|  |  |  |  |  |  |

M) Standard deviation of secondary structure prediction for GOR method using Five-fold cross-validation, with the consideration of actual two-state RSA information.

|  |  | Thresholds | | | | | |
| --- | --- | --- | --- | --- | --- | --- | --- |
|  |  | 4 | 9 | 16 | 25 | 36 | 50 |
| Total |  | 1.471 | 0.779 | 0.642 | 0.854 | 0.526 | 1.144 |
|  |  |  |  |  |  |  |  |
| A |  | 2.097 | 0.333 | 0.550 | 0.576 | 0.548 | 0.924 |
| C |  | 1.795 | 0.783 | 1.198 | 0.767 | 0.442 | 0.257 |
| D |  | 1.676 | 0.542 | 0.374 | 0.178 | 0.353 | 0.172 |
| E |  | 1.596 | 0.275 | 0.340 | 0.490 | 0.649 | 1.001 |
| F |  | 1.093 | 0.258 | 0.548 | 0.827 | 0.717 | 0.622 |
| G |  | 1.773 | 0.216 | 0.378 | 0.318 | 0.319 | 0.212 |
| H |  | 1.553 | 0.454 | 0.806 | 0.789 | 0.441 | 0.139 |
| I |  | 1.743 | 1.204 | 0.952 | 0.505 | 0.444 | 0.237 |
| K |  | 1.699 | 0.493 | 0.686 | 0.852 | 1.039 | 0.272 |
| L |  | 1.799 | 0.743 | 0.889 | 0.624 | 0.178 | 0.123 |
| M |  | 1.256 | 0.237 | 0.389 | 0.328 | 0.257 | 0.214 |
| N |  | 1.662 | 0.470 | 0.111 | 0.144 | 0.240 | 0.193 |
| P |  | 1.833 | 0.588 | 1.037 | 0.416 | 0.281 | 0.272 |
| Q |  | 2.003 | 0.328 | 0.334 | 0.150 | 0.639 | 1.350 |
| R |  | 1.685 | 0.225 | 0.213 | 0.413 | 0.895 | 1.116 |
| S |  | 1.408 | 0.374 | 0.043 | 0.248 | 0.350 | 0.176 |
| T |  | 1.418 | 0.787 | 0.772 | 0.208 | 0.376 | 0.403 |
| V |  | 1.133 | 0.908 | 0.492 | 0.461 | 0.408 | 0.646 |
| W |  | 0.790 | 0.452 | 0.618 | 0.678 | 0.690 | 0.613 |
| Y |  | 0.152 | 0.037 | 0.044 | 0.024 | 0.027 | 0.037 |
|  |  |  |  |  |  |  |  |

N) Standard deviation of secondary structure prediction for GOR method using Five-fold cross-validation, with the consideration of predicted two-state RSA information.

|  |  | Thresholds | | | | | |
| --- | --- | --- | --- | --- | --- | --- | --- |
|  |  | 4 | 9 | 16 | 25 | 36 | 50 |
| Total |  | 2.097 | 0.833 | 0.550 | 0.576 | 0.548 | 0.924 |
|  |  |  |  |  |  |  |  |
| A |  | 1.471 | 0.179 | 0.142 | 0.154 | 0.326 | 1.144 |
| C |  | 0.466 | 0.294 | 0.811 | 0.635 | 0.414 | 0.406 |
| D |  | 1.434 | 0.136 | 0.141 | 0.074 | 0.079 | 0.069 |
| E |  | 1.428 | 0.295 | 0.321 | 0.527 | 0.769 | 0.884 |
| F |  | 0.606 | 0.160 | 0.340 | 0.494 | 0.353 | 0.454 |
| G |  | 1.705 | 0.174 | 0.191 | 0.113 | 0.135 | 0.055 |
| H |  | 0.857 | 0.546 | 0.781 | 0.572 | 0.220 | 0.151 |
| I |  | 1.020 | 0.515 | 0.343 | 0.202 | 0.249 | 0.168 |
| K |  | 0.621 | 0.443 | 0.575 | 0.706 | 0.872 | 0.953 |
| L |  | 0.848 | 0.395 | 0.419 | 0.397 | 0.219 | 0.218 |
| M |  | 1.019 | 0.305 | 0.253 | 0.175 | 0.235 | 0.335 |
| N |  | 1.551 | 0.197 | 0.154 | 0.060 | 0.133 | 0.060 |
| P |  | 1.918 | 0.115 | 0.086 | 0.033 | 0.047 | 0.068 |
| Q |  | 1.453 | 0.420 | 0.337 | 0.201 | 0.577 | 1.410 |
| R |  | 1.528 | 0.442 | 0.348 | 0.270 | 0.765 | 1.267 |
| S |  | 1.274 | 0.367 | 0.155 | 0.134 | 0.076 | 0.090 |
| T |  | 0.729 | 0.692 | 0.648 | 0.295 | 0.252 | 0.291 |
| V |  | 1.688 | 0.453 | 0.363 | 0.300 | 0.280 | 0.226 |
| W |  | 0.630 | 0.489 | 0.318 | 0.342 | 0.489 | 0.403 |
| Y |  | 0.119 | 0.013 | 0.018 | 0.015 | 0.023 | 0.055 |
|  |  |  |  |  |  |  |  |

O) Standard deviation of secondary structure prediction for GOR method using Five-fold cross-validation, with the consideration of actual three-state RSA information.

|  |  | Thresholds | | | |
| --- | --- | --- | --- | --- | --- |
|  |  | [4,16] | [9,16] | [9,36] | [16,36] |
| Total |  | 1.498 | 0.683 | 0.493 | 0.636 |
|  |  |  |  |  |  |
| A |  | 1.766 | 1.144 | 0.356 | 1.155 |
| C |  | 0.287 | 1.115 | 0.367 | 0.262 |
| D |  | 1.306 | 0.255 | 1.008 | 0.369 |
| E |  | 1.399 | 0.420 | 0.242 | 0.614 |
| F |  | 0.350 | 0.379 | 0.672 | 1.171 |
| G |  | 1.179 | 0.380 | 0.221 | 0.837 |
| H |  | 1.968 | 1.329 | 0.856 | 1.252 |
| I |  | 1.486 | 0.577 | 1.078 | 0.389 |
| K |  | 1.185 | 0.716 | 0.264 | 0.556 |
| L |  | 0.783 | 0.697 | 0.836 | 0.715 |
| M |  | 0.264 | 0.307 | 0.300 | 0.285 |
| N |  | 0.141 | 0.361 | 0.799 | 0.398 |
| P |  | 1.575 | 0.400 | 0.541 | 0.317 |
| Q |  | 1.608 | 0.308 | 0.835 | 0.221 |
| R |  | 0.446 | 0.447 | 0.046 | 0.060 |
| S |  | 1.116 | 0.716 | 0.224 | 0.797 |
| T |  | 0.852 | 0.810 | 0.280 | 0.569 |
| V |  | 0.937 | 0.587 | 0.540 | 0.403 |
| W |  | 1.302 | 0.630 | 0.282 | 0.632 |
| Y |  | 0.083 | 0.049 | 0.022 | 0.047 |
|  |  |  |  |  |  |

P) Standard deviation of secondary structure prediction for GOR method using Five-fold cross-validation, with the consideration of predicted three-state RSA information.

|  |  | Thresholds | | | |
| --- | --- | --- | --- | --- | --- |
|  |  | [4,16] | [9,16] | [9,36] | [16,36] |
| Total |  | 1.471 | 0.513 | 0.588 | 1.177 |
|  |  |  |  |  |  |
| A |  | 0.466 | 1.053 | 0.401 | 1.160 |
| C |  | 1.434 | 0.202 | 0.312 | 0.261 |
| D |  | 1.428 | 0.338 | 0.975 | 0.422 |
| E |  | 0.606 | 0.358 | 0.377 | 0.708 |
| F |  | 1.705 | 0.211 | 0.349 | 0.277 |
| G |  | 0.857 | 0.569 | 0.278 | 0.978 |
| H |  | 1.020 | 1.397 | 0.483 | 1.237 |
| I |  | 0.621 | 0.387 | 1.064 | 0.313 |
| K |  | 0.848 | 0.161 | 0.428 | 0.251 |
| L |  | 1.019 | 0.241 | 0.390 | 0.555 |
| M |  | 1.551 | 0.198 | 0.207 | 0.412 |
| N |  | 1.918 | 0.307 | 0.201 | 0.404 |
| P |  | 1.453 | 0.370 | 0.447 | 0.289 |
| Q |  | 1.528 | 0.278 | 0.622 | 0.268 |
| R |  | 1.274 | 0.523 | 0.043 | 0.063 |
| S |  | 0.729 | 0.754 | 0.252 | 0.857 |
| T |  | 1.688 | 0.554 | 0.200 | 0.256 |
| V |  | 0.630 | 0.225 | 0.309 | 0.430 |
| W |  | 0.409 | 0.617 | 0.271 | 0.496 |
| Y |  | 0.119 | 0.043 | 0.012 | 0.041 |
|  |  |  |  |  |  |

Q) Standard deviation of secondary structure prediction for GOR method using Five-fold cross-validation, with the consideration of residue-specific classification of actual RSA information.

|  |  | Thresholds | | | |
| --- | --- | --- | --- | --- | --- |
|  |  | Tertiles | Mean±SD | Mean | Median |
| Total |  | 1.269 | 0.961 | 0.548 | 0.465 |
|  |  |  |  |  |  |
| A |  | 1.680 | 0.911 | 1.163 | 0.448 |
| C |  | 0.365 | 0.195 | 0.339 | 0.259 |
| D |  | 0.993 | 0.448 | 0.326 | 0.221 |
| E |  | 0.431 | 0.319 | 1.177 | 0.210 |
| F |  | 1.385 | 0.212 | 0.050 | 0.235 |
| G |  | 0.365 | 0.258 | 1.107 | 0.283 |
| H |  | 1.516 | 0.706 | 1.391 | 0.259 |
| I |  | 0.975 | 0.554 | 1.031 | 0.199 |
| K |  | 0.965 | 0.485 | 0.469 | 0.158 |
| L |  | 0.834 | 0.316 | 1.004 | 0.267 |
| M |  | 0.503 | 0.369 | 0.347 | 0.342 |
| N |  | 0.433 | 0.304 | 0.894 | 0.212 |
| P |  | 0.781 | 0.411 | 0.800 | 0.220 |
| Q |  | 0.768 | 0.322 | 0.616 | 0.207 |
| R |  | 0.723 | 0.335 | 0.086 | 0.217 |
| S |  | 0.378 | 0.341 | 0.885 | 0.048 |
| T |  | 1.098 | 0.669 | 1.033 | 0.202 |
| V |  | 0.539 | 0.395 | 0.786 | 0.191 |
| W |  | 1.083 | 0.633 | 0.302 | 0.629 |
| Y |  | 0.053 | 0.022 | 0.055 | 0.011 |
|  |  |  |  |  |  |

R) Standard deviation of secondary structure prediction for GOR method using Five-fold cross-validation, with the consideration of residue-specific classification of predicted RSA information.

|  |  | Thresholds | | | |
| --- | --- | --- | --- | --- | --- |
|  |  | Tertiles | Mean±SD | Mean | Median |
| Total |  | 0.240 | 2.264 | 0.426 | 0.451 |
|  |  |  |  |  |  |
| A |  | 0.225 | 0.865 | 1.481 | 1.102 |
| C |  | 0.279 | 0.075 | 0.277 | 0.197 |
| D |  | 0.443 | 2.160 | 1.403 | 0.362 |
| E |  | 0.231 | 0.806 | 0.705 | 0.163 |
| F |  | 0.331 | 0.220 | 0.312 | 0.237 |
| G |  | 0.154 | 0.477 | 0.591 | 0.284 |
| H |  | 0.194 | 2.173 | 1.521 | 1.342 |
| I |  | 0.138 | 2.299 | 0.514 | 0.360 |
| K |  | 0.348 | 1.878 | 0.504 | 1.131 |
| L |  | 0.105 | 2.204 | 0.191 | 0.485 |
| M |  | 0.296 | 0.105 | 0.195 | 0.271 |
| N |  | 0.244 | 0.109 | 0.270 | 0.275 |
| P |  | 0.389 | 2.213 | 1.629 | 0.425 |
| Q |  | 0.299 | 2.278 | 1.612 | 0.706 |
| R |  | 0.024 | 0.552 | 0.264 | 0.268 |
| S |  | 0.126 | 0.187 | 0.455 | 0.047 |
| T |  | 0.196 | 1.141 | 0.451 | 0.289 |
| V |  | 0.135 | 2.068 | 0.238 | 0.360 |
| W |  | 0.965 | 1.136 | 0.299 | 0.065 |
| Y |  | 0.020 | 0.151 | 0.046 | 0.055 |
|  |  |  |  |  |  |
