# Supplementary material for: Impact of residue accessible surface area on the prediction of protein secondary structures
Source: BMC Bioinformatics. 2008 Aug 31;9:357. doi: 10.1186/1471-2105-9-357 (PMC2553345; doi:10.1186/1471-2105-9-357)
Supplement: Additional file 3 — Accuracy of secondary structure prediction for Chou-Fasman method, with the consideration of actual and predicted RSA information. [file 1471-2105-9-357-S3.doc]

A) Accuracy of secondary structure prediction for Chou-Fasman method using leave-one-out cross-validation, with the consideration of actual two-state RSA information. Totally, 1571044 residues were present in the assessed dataset. For each of the twenty amino acids the accuracy of prediction is reported separately.

|  |  | Thresholds | | | | | |
| --- | --- | --- | --- | --- | --- | --- | --- |
|  |  | 4 | 9 | 16 | 25 | 36 | 50 |
| Correct |  | 704628 | 728790 | 749778 | 731422 | 709179 | 707669 |
| False |  | 866416 | 842254 | 821266 | 839622 | 861865 | 863375 |
| Q3 |  | 46.780 | 48.114 | 48.260 | 48.190 | 46.619 | 46.946 |
| SD |  | 9.813 | 9.458 | 8.673 | 9.758 | 9.928 | 9.993 |
|  |  |  |  |  |  |  |  |
| A |  | 48.767 | 49.814 | 54.226 | 52.251 | 51.876 | 49.950 |
| C |  | 46.053 | 47.739 | 42.020 | 43.748 | 43.771 | 45.134 |
| D |  | 41.860 | 44.653 | 46.945 | 43.730 | 41.799 | 42.615 |
| E |  | 48.842 | 49.744 | 52.866 | 50.345 | 50.647 | 48.709 |
| F |  | 43.266 | 44.117 | 44.090 | 45.081 | 44.519 | 44.410 |
| G |  | 41.091 | 45.372 | 48.058 | 43.246 | 40.922 | 38.833 |
| H |  | 43.627 | 45.247 | 47.901 | 42.505 | 40.217 | 42.168 |
| I |  | 47.687 | 48.087 | 46.655 | 49.547 | 48.292 | 47.779 |
| K |  | 44.658 | 45.572 | 47.500 | 45.634 | 43.922 | 44.490 |
| L |  | 47.904 | 48.751 | 47.780 | 49.157 | 47.787 | 47.531 |
| M |  | 44.415 | 45.285 | 43.787 | 46.432 | 44.872 | 45.118 |
| N |  | 41.287 | 44.699 | 47.258 | 43.462 | 41.502 | 42.391 |
| P |  | 44.255 | 48.078 | 49.335 | 45.178 | 42.968 | 44.392 |
| Q |  | 45.663 | 46.693 | 49.959 | 47.229 | 47.889 | 45.575 |
| R |  | 44.307 | 45.236 | 48.060 | 45.689 | 44.158 | 43.909 |
| S |  | 42.506 | 44.976 | 46.789 | 40.514 | 37.319 | 40.772 |
| T |  | 38.390 | 39.527 | 39.714 | 43.535 | 40.900 | 42.943 |
| V |  | 49.330 | 49.601 | 48.633 | 51.477 | 50.336 | 49.402 |
| W |  | 41.943 | 41.028 | 42.053 | 43.983 | 43.069 | 42.039 |
| Y |  | 41.771 | 42.271 | 43.101 | 44.891 | 43.542 | 44.112 |
|  |  |  |  |  |  |  |  |

B) Accuracy of secondary structure prediction for Chou-Fasman method using leave-one-out cross-validation, with the consideration of predicted two-state RSA information.

|  |  | Thresholds | | | | | |
| --- | --- | --- | --- | --- | --- | --- | --- |
|  |  | 4 | 9 | 16 | 25 | 36 | 50 |
| Correct |  | 418787 | 738567 | 789814 | 812434 | 660788 | 630406 |
| False |  | 1152257 | 832477 | 781230 | 758610 | 910256 | 940638 |
| Q3 |  | 28.437 | 47.661 | 50.578 | 51.827 | 43.044 | 41.088 |
| SD |  | 14.442 | 8.799 | 8.546 | 8.987 | 10.043 | 10.644 |
|  |  |  |  |  |  |  |  |
| A |  | 23.945 | 52.258 | 54.629 | 56.325 | 46.681 | 47.109 |
| C |  | 31.775 | 42.233 | 44.441 | 45.549 | 39.586 | 39.951 |
| D |  | 18.790 | 46.610 | 50.939 | 53.052 | 39.628 | 35.811 |
| E |  | 22.269 | 51.454 | 54.155 | 55.575 | 48.881 | 46.517 |
| F |  | 32.778 | 42.915 | 45.842 | 46.464 | 40.836 | 40.667 |
| G |  | 20.064 | 48.688 | 53.833 | 56.364 | 35.251 | 32.837 |
| H |  | 25.946 | 47.870 | 50.116 | 51.320 | 36.540 | 33.456 |
| I |  | 39.415 | 45.850 | 46.782 | 47.415 | 45.851 | 45.247 |
| K |  | 22.892 | 46.738 | 49.116 | 50.342 | 43.748 | 40.684 |
| L |  | 28.955 | 46.195 | 48.942 | 50.352 | 45.099 | 44.095 |
| M |  | 26.178 | 40.852 | 45.001 | 46.834 | 39.178 | 39.112 |
| N |  | 19.248 | 46.763 | 51.038 | 53.356 | 38.690 | 35.203 |
| P |  | 19.726 | 49.165 | 55.085 | 57.795 | 39.940 | 36.523 |
| Q |  | 22.561 | 48.442 | 50.816 | 52.303 | 43.867 | 42.579 |
| R |  | 24.225 | 46.647 | 48.897 | 50.160 | 42.015 | 39.487 |
| S |  | 23.499 | 47.039 | 49.968 | 51.176 | 34.644 | 30.262 |
| T |  | 28.450 | 40.266 | 49.170 | 49.823 | 38.326 | 34.813 |
| V |  | 43.278 | 48.192 | 49.170 | 49.316 | 47.705 | 46.929 |
| W |  | 30.612 | 42.641 | 45.646 | 46.680 | 39.994 | 39.232 |
| Y |  | 32.309 | 42.778 | 45.549 | 47.671 | 39.879 | 39.198 |
|  |  |  |  |  |  |  |  |

|  |  | Thresholds | | | |
| --- | --- | --- | --- | --- | --- |
|  |  | [4,16] | [9,16] | [9,36] | [16,36] |
| Correct |  | 739860 | 721811 | 705423 | 709116 |
| False |  | 831184 | 849233 | 865621 | 861928 |
| Q3 |  | 47.094 | 45.945 | 44.902 | 47.117 |
| SD |  | 9.759 | 10.023 | 10.369 | 10.154 |
|  |  |  |  |  |  |
| A |  | 49.435 | 48.884 | 47.947 | 48.741 |
| C |  | 47.642 | 45.864 | 45.568 | 45.337 |
| D |  | 45.752 | 43.605 | 41.664 | 41.628 |
| E |  | 49.420 | 48.888 | 48.015 | 48.939 |
| F |  | 46.515 | 45.567 | 44.737 | 45.101 |
| G |  | 46.802 | 43.462 | 41.079 | 40.453 |
| H |  | 44.550 | 43.505 | 41.097 | 43.078 |
| I |  | 48.062 | 48.042 | 48.850 | 48.106 |
| K |  | 45.614 | 44.866 | 43.581 | 44.463 |
| L |  | 48.724 | 47.987 | 47.604 | 47.707 |
| M |  | 46.931 | 46.248 | 45.229 | 45.652 |
| N |  | 45.408 | 42.874 | 41.041 | 40.600 |
| P |  | 49.583 | 46.718 | 44.206 | 44.143 |
| Q |  | 46.156 | 45.485 | 44.939 | 45.693 |
| R |  | 44.674 | 44.239 | 42.951 | 44.327 |
| S |  | 45.634 | 43.791 | 42.426 | 41.879 |
| T |  | 45.164 | 44.374 | 42.546 | 43.688 |
| V |  | 49.900 | 49.686 | 49.801 | 49.819 |
| W |  | 42.990 | 42.650 | 42.614 | 42.531 |
| Y |  | 43.391 | 42.833 | 43.398 | 42.212 |
|  |  |  |  |  |  |

C) Accuracy of secondary structure prediction for Chou-Fasman method using leave-one-out cross-validation, with the consideration of actual three-state RSA information.

D) Accuracy of secondary structure prediction for Chou-Fasman method using leave-one-out cross-validation, with the consideration of predicted three-state RSA information.

|  |  | Thresholds | | | |
| --- | --- | --- | --- | --- | --- |
|  |  | [4,16] | [9,16] | [9,36] | [16,36] |
| Correct |  | 799079 | 753038 | 690996 | 677547 |
| False |  | 771965 | 818006 | 880048 | 893497 |
| Q3 |  | 51.170 | 48.452 | 44.884 | 44.157 |
| SD |  | 8.659 | 8.626 | 9.620 | 10.026 |
|  |  |  |  |  |  |
| A |  | 54.550 | 50.923 | 48.091 | 44.875 |
| C |  | 46.016 | 42.843 | 41.498 | 41.037 |
| D |  | 52.674 | 48.650 | 43.018 | 41.386 |
| E |  | 54.981 | 51.962 | 49.083 | 48.634 |
| F |  | 46.194 | 43.246 | 41.594 | 41.307 |
| G |  | 55.756 | 51.242 | 42.833 | 39.380 |
| H |  | 49.389 | 47.624 | 38.230 | 42.156 |
| I |  | 46.366 | 45.161 | 45.090 | 45.544 |
| K |  | 50.113 | 47.245 | 44.598 | 43.757 |
| L |  | 49.563 | 45.872 | 44.205 | 44.403 |
| M |  | 44.214 | 40.419 | 37.632 | 38.093 |
| N |  | 52.460 | 48.771 | 42.640 | 40.991 |
| P |  | 57.345 | 52.519 | 45.459 | 42.999 |
| Q |  | 50.830 | 48.196 | 44.740 | 44.242 |
| R |  | 49.071 | 46.802 | 41.971 | 42.203 |
| S |  | 50.944 | 48.341 | 42.846 | 41.362 |
| T |  | 49.605 | 47.802 | 39.999 | 39.547 |
| V |  | 49.187 | 48.004 | 47.480 | 47.727 |
| W |  | 44.102 | 42.522 | 39.815 | 40.573 |
| Y |  | 44.628 | 43.210 | 40.725 | 40.023 |
|  |  |  |  |  |  |

E) Accuracy of secondary structure prediction for Chou-Fasman method using leave-one-out cross-validation, with the consideration of residue-specific classification of actual RSA information.

|  |  | Thresholds | | | |
| --- | --- | --- | --- | --- | --- |
|  |  | Tertiles | Mean±SD | Mean | Median |
| Correct |  | 854368 | 855984 | 850542 | 830125 |
| False |  | 716676 | 715060 | 720502 | 740919 |
| Q3 |  | 54.382 | 54.485 | 54.139 | 52.839 |
| SD |  | 9.952 | 9.912 | 10.373 | 10.378 |
|  |  |  |  |  |  |
| A |  | 59.925 | 60.274 | 59.664 | 58.209 |
| C |  | 46.459 | 45.582 | 47.148 | 45.092 |
| D |  | 53.377 | 52.925 | 51.498 | 50.540 |
| E |  | 58.252 | 57.388 | 57.070 | 56.463 |
| F |  | 43.982 | 42.329 | 43.889 | 43.816 |
| G |  | 47.428 | 46.945 | 47.246 | 44.861 |
| H |  | 54.549 | 53.920 | 53.230 | 54.927 |
| I |  | 48.108 | 48.713 | 48.611 | 47.175 |
| K |  | 51.460 | 51.827 | 51.565 | 49.014 |
| L |  | 48.124 | 47.890 | 48.485 | 48.608 |
| M |  | 46.324 | 43.787 | 44.765 | 44.196 |
| N |  | 47.329 | 47.382 | 47.308 | 47.639 |
| P |  | 54.252 | 56.448 | 53.853 | 51.616 |
| Q |  | 55.096 | 55.348 | 55.040 | 53.766 |
| R |  | 52.527 | 52.953 | 52.630 | 51.278 |
| S |  | 50.966 | 53.958 | 52.475 | 48.758 |
| T |  | 42.369 | 42.457 | 43.528 | 42.015 |
| V |  | 49.760 | 49.834 | 49.801 | 49.420 |
| W |  | 42.264 | 42.494 | 42.568 | 42.421 |
| Y |  | 44.050 | 44.214 | 44.031 | 43.978 |
|  |  |  |  |  |  |

F) Accuracy of secondary structure prediction for Chou-Fasman method using leave-one-out cross-validation, with the consideration of residue-specific classification of predicted RSA information.

|  |  | Thresholds | | | |
| --- | --- | --- | --- | --- | --- |
|  |  | Tertiles | Mean±SD | Mean | Median |
| Correct |  | 570704 | 387505 | 799607 | 829048 |
| False |  | 1000340 | 1183539 | 771437 | 741996 |
| Q3 |  | 37.987 | 26.150 | 52.052 | 53.381 |
| SD |  | 12.537 | 15.061 | 9.019 | 8.616 |
|  |  |  |  |  |  |
| A |  | 35.930 | 19.455 | 55.565 | 56.857 |
| C |  | 41.850 | 33.429 | 49.554 | 50.861 |
| D |  | 30.652 | 15.333 | 49.711 | 52.498 |
| E |  | 31.823 | 17.680 | 53.397 | 54.858 |
| F |  | 40.081 | 32.908 | 49.463 | 50.658 |
| G |  | 35.745 | 18.747 | 52.558 | 57.656 |
| H |  | 34.472 | 24.794 | 46.680 | 48.518 |
| I |  | 44.157 | 39.502 | 51.402 | 51.673 |
| K |  | 33.039 | 19.971 | 48.948 | 50.958 |
| L |  | 36.673 | 26.719 | 52.372 | 53.218 |
| M |  | 35.466 | 25.165 | 50.178 | 51.710 |
| N |  | 31.981 | 16.881 | 50.089 | 53.538 |
| P |  | 35.297 | 14.824 | 54.614 | 59.642 |
| Q |  | 32.850 | 19.446 | 50.896 | 52.745 |
| R |  | 33.450 | 21.886 | 49.181 | 50.957 |
| S |  | 33.325 | 21.913 | 46.027 | 48.935 |
| T |  | 37.459 | 28.832 | 47.192 | 49.027 |
| V |  | 47.173 | 43.513 | 52.471 | 51.831 |
| W |  | 37.100 | 30.134 | 47.935 | 47.994 |
| Y |  | 38.697 | 32.461 | 47.943 | 48.244 |
|  |  |  |  |  |  |

G) Accuracy of secondary structure prediction for Chou-Fasman method using Five-fold cross-validation, with the consideration of actual two-state RSA information.

|  |  | Thresholds | | | | | |
| --- | --- | --- | --- | --- | --- | --- | --- |
|  |  | 4 | 9 | 16 | 25 | 36 | 50 |
| Correct |  | 719926.687 | 740029.784 | 755842.316 | 740162.899 | 723311.059 | 722499.132 |
| False |  | 851117.313 | 831014.216 | 815201.684 | 830881.101 | 847732.941 | 848544.868 |
| Q3 |  | 45.825 | 47.104 | 48.111 | 47.113 | 46.040 | 45.988 |
|  |  |  |  |  |  |  |  |
| A |  | 49.750 | 50.709 | 54.185 | 52.719 | 52.644 | 51.274 |
| C |  | 46.621 | 48.177 | 43.296 | 44.436 | 44.371 | 45.331 |
| D |  | 42.929 | 45.145 | 46.794 | 44.259 | 42.751 | 43.389 |
| E |  | 49.719 | 50.647 | 53.117 | 51.022 | 51.286 | 49.969 |
| F |  | 44.126 | 44.844 | 45.023 | 45.795 | 45.283 | 45.229 |
| G |  | 42.535 | 45.693 | 47.513 | 43.310 | 41.766 | 40.237 |
| H |  | 44.624 | 45.923 | 47.855 | 43.361 | 41.443 | 42.803 |
| I |  | 48.487 | 48.939 | 47.781 | 50.082 | 49.236 | 48.800 |
| K |  | 45.423 | 46.361 | 47.802 | 46.252 | 44.858 | 45.291 |
| L |  | 48.647 | 49.588 | 48.716 | 49.728 | 48.651 | 48.490 |
| M |  | 45.084 | 46.032 | 44.793 | 46.946 | 45.764 | 45.938 |
| N |  | 42.538 | 45.108 | 46.864 | 43.944 | 42.454 | 43.146 |
| P |  | 45.497 | 48.499 | 49.258 | 45.848 | 44.037 | 45.166 |
| Q |  | 46.568 | 47.546 | 50.063 | 47.840 | 48.400 | 46.827 |
| R |  | 45.171 | 46.031 | 48.225 | 46.220 | 45.082 | 44.856 |
| S |  | 43.605 | 45.538 | 46.758 | 41.501 | 38.710 | 41.379 |
| T |  | 39.249 | 40.160 | 40.947 | 44.190 | 41.987 | 43.427 |
| V |  | 50.191 | 50.507 | 49.758 | 52.033 | 51.173 | 50.524 |
| W |  | 42.460 | 41.922 | 42.897 | 44.393 | 43.799 | 43.004 |
| Y |  | 42.625 | 43.075 | 43.761 | 45.304 | 44.394 | 44.839 |
|  |  |  |  |  |  |  |  |

H) Accuracy of secondary structure prediction for Chou-Fasman method using Five-fold cross-validation, with the consideration of predicted two-state RSA information.

|  |  | Thresholds | | | | | |
| --- | --- | --- | --- | --- | --- | --- | --- |
|  |  | 4 | 9 | 16 | 25 | 36 | 50 |
| Correct |  | 424676.503 | 604644.054 | 799577.398 | 758705.245 | 708818.356 | 693100.065 |
| False |  | 1146367.497 | 966399.946 | 771466.602 | 812338.755 | 862225.644 | 877943.935 |
| Q3 |  | 27.031 | 38.487 | 50.895 | 48.293 | 45.118 | 44.117 |
|  |  |  |  |  |  |  |  |
| A |  | 22.363 | 38.560 | 54.440 | 51.257 | 49.193 | 46.119 |
| C |  | 34.741 | 43.351 | 46.600 | 43.890 | 42.945 | 42.178 |
| D |  | 18.844 | 33.330 | 51.973 | 48.379 | 43.703 | 42.057 |
| E |  | 20.812 | 34.768 | 54.664 | 52.022 | 50.038 | 49.526 |
| F |  | 33.919 | 41.425 | 46.845 | 44.259 | 43.034 | 42.466 |
| G |  | 22.706 | 38.394 | 54.846 | 50.711 | 43.479 | 40.125 |
| H |  | 27.029 | 36.650 | 49.266 | 47.708 | 39.616 | 43.022 |
| I |  | 40.057 | 45.360 | 47.571 | 46.509 | 46.722 | 46.778 |
| K |  | 22.551 | 35.389 | 49.968 | 47.376 | 45.499 | 44.579 |
| L |  | 28.698 | 38.861 | 49.957 | 46.774 | 45.680 | 45.630 |
| M |  | 26.963 | 37.565 | 44.742 | 41.387 | 39.364 | 39.572 |
| N |  | 20.456 | 34.662 | 51.775 | 48.458 | 43.309 | 41.627 |
| P |  | 19.242 | 38.208 | 56.498 | 52.213 | 46.213 | 43.771 |
| Q |  | 22.336 | 35.402 | 50.678 | 48.365 | 45.782 | 45.183 |
| R |  | 24.428 | 35.792 | 48.964 | 46.972 | 43.089 | 43.157 |
| S |  | 24.666 | 35.645 | 50.668 | 48.396 | 43.779 | 42.189 |
| T |  | 30.590 | 39.240 | 49.649 | 48.027 | 41.304 | 40.585 |
| V |  | 43.885 | 48.107 | 50.512 | 49.430 | 49.190 | 48.961 |
| W |  | 31.185 | 38.501 | 44.692 | 43.265 | 41.190 | 41.631 |
| Y |  | 33.443 | 40.024 | 45.292 | 44.009 | 42.122 | 41.208 |
|  |  |  |  |  |  |  |  |

I) Accuracy of secondary structure prediction for Chou-Fasman method using Five-fold cross-validation, with the consideration of actual three-state RSA information.

|  |  | Thresholds | | | |
| --- | --- | --- | --- | --- | --- |
|  |  | [4,16] | [9,16] | [9,36] | [16,36] |
| Correct |  | 775826.325 | 758844.223 | 748243.852 | 721639.162 |
| False |  | 795217.675 | 812199.777 | 822800.148 | 849404.838 |
| Q3 |  | 49.383 | 48.302 | 47.627 | 45.934 |
|  |  |  |  |  |  |
| A |  | 50.630 | 49.731 | 49.090 | 49.584 |
| C |  | 48.003 | 46.543 | 46.399 | 45.981 |
| D |  | 46.421 | 44.418 | 42.683 | 42.264 |
| E |  | 50.468 | 49.751 | 49.135 | 49.794 |
| F |  | 47.210 | 46.262 | 45.651 | 45.794 |
| G |  | 47.409 | 44.372 | 42.174 | 41.152 |
| H |  | 45.206 | 44.263 | 42.290 | 43.835 |
| I |  | 48.970 | 48.744 | 49.707 | 49.102 |
| K |  | 46.429 | 45.606 | 44.586 | 45.161 |
| L |  | 49.600 | 48.750 | 48.566 | 48.587 |
| M |  | 47.516 | 46.726 | 46.084 | 46.297 |
| N |  | 46.085 | 43.696 | 42.051 | 41.298 |
| P |  | 50.150 | 47.614 | 45.369 | 44.796 |
| Q |  | 47.219 | 46.308 | 45.948 | 46.466 |
| R |  | 45.543 | 44.975 | 43.941 | 44.984 |
| S |  | 46.136 | 44.628 | 43.443 | 42.696 |
| T |  | 45.701 | 45.023 | 43.425 | 44.171 |
| V |  | 50.863 | 50.462 | 50.762 | 50.791 |
| W |  | 43.783 | 43.279 | 43.467 | 43.435 |
| Y |  | 44.267 | 43.552 | 44.222 | 43.285 |
|  |  |  |  |  |  |

J) Accuracy of secondary structure prediction for Chou-Fasman method using Five-fold cross-validation, with the consideration of predicted three-state RSA information.

|  |  | Thresholds | | | |
| --- | --- | --- | --- | --- | --- |
|  |  | [4,16] | [9,16] | [9,36] | [16,36] |
| Correct |  | 799577.398 | 758705.245 | 708818.356 | 693100.065 |
| False |  | 771466.602 | 812338.755 | 862225.644 | 877943.935 |
| Q3 |  | 50.895 | 48.293 | 45.118 | 44.117 |
|  |  |  |  |  |  |
| A |  | 54.440 | 51.257 | 49.193 | 46.119 |
| C |  | 46.600 | 43.890 | 42.945 | 42.178 |
| D |  | 51.973 | 48.379 | 43.703 | 42.057 |
| E |  | 54.664 | 52.022 | 50.038 | 49.526 |
| F |  | 46.845 | 44.259 | 43.034 | 42.466 |
| G |  | 54.846 | 50.711 | 43.479 | 40.125 |
| H |  | 49.266 | 47.708 | 39.616 | 43.022 |
| I |  | 47.571 | 46.509 | 46.722 | 46.778 |
| K |  | 49.968 | 47.376 | 45.499 | 44.579 |
| L |  | 49.957 | 46.774 | 45.680 | 45.630 |
| M |  | 44.742 | 41.387 | 39.364 | 39.572 |
| N |  | 51.775 | 48.458 | 43.309 | 41.627 |
| P |  | 56.498 | 52.213 | 46.213 | 43.771 |
| Q |  | 50.678 | 48.365 | 45.782 | 45.183 |
| R |  | 48.964 | 46.972 | 43.089 | 43.157 |
| S |  | 50.668 | 48.396 | 43.779 | 42.189 |
| T |  | 49.649 | 48.027 | 41.304 | 40.585 |
| V |  | 50.512 | 49.430 | 49.190 | 48.961 |
| W |  | 44.692 | 43.265 | 41.190 | 41.631 |
| Y |  | 45.292 | 44.009 | 42.122 | 41.208 |
|  |  |  |  |  |  |

K) Accuracy of secondary structure prediction for Chou-Fasman method using Five-fold cross-validation, with the consideration of residue-specific classification of actual RSA information.

|  |  | Thresholds | | | |
| --- | --- | --- | --- | --- | --- |
|  |  | Tertiles | Mean±SD | Mean | Median |
| Correct |  | 850561.062 | 853653.276 | 848097.355 | 838001.369 |
| False |  | 720482.938 | 717390.724 | 722946.645 | 733042.631 |
| Q3 |  | 54.140 | 54.337 | 53.983 | 53.340 |
|  |  |  |  |  |  |
| A |  | 58.997 | 59.268 | 58.442 | 57.371 |
| C |  | 47.251 | 46.173 | 47.568 | 46.308 |
| D |  | 52.022 | 51.845 | 50.281 | 49.796 |
| E |  | 57.388 | 56.766 | 56.330 | 55.915 |
| F |  | 45.097 | 43.578 | 45.032 | 44.693 |
| G |  | 47.519 | 47.386 | 46.379 | 44.794 |
| H |  | 52.936 | 52.133 | 51.777 | 53.258 |
| I |  | 49.197 | 49.320 | 49.524 | 48.368 |
| K |  | 51.116 | 51.567 | 50.829 | 48.801 |
| L |  | 49.093 | 48.623 | 49.265 | 49.492 |
| M |  | 47.173 | 44.772 | 45.588 | 45.177 |
| N |  | 47.118 | 47.433 | 46.850 | 47.429 |
| P |  | 53.590 | 55.490 | 52.649 | 51.204 |
| Q |  | 54.267 | 54.468 | 53.929 | 52.927 |
| R |  | 51.915 | 52.205 | 51.728 | 50.633 |
| S |  | 50.508 | 53.034 | 51.045 | 48.143 |
| T |  | 43.354 | 43.189 | 44.157 | 43.142 |
| V |  | 50.922 | 50.705 | 50.764 | 50.469 |
| W |  | 43.194 | 43.182 | 43.311 | 43.203 |
| Y |  | 44.849 | 45.011 | 44.531 | 44.512 |
|  |  |  |  |  |  |

L) Accuracy of secondary structure prediction for Chou-Fasman method using Five-fold cross-validation, with the consideration of residue-specific classification of predicted RSA information.

|  |  | Thresholds | | | |
| --- | --- | --- | --- | --- | --- |
|  |  | Tertiles | Mean±SD | Mean | Median |
| Correct |  | 604644.054 | 424676.503 | 803597.846 | 829787.848 |
| False |  | 966399.946 | 1146367.497 | 767446.154 | 741256.152 |
| Q3 |  | 38.487 | 27.031 | 51.151 | 52.818 |
|  |  |  |  |  |  |
| A |  | 38.560 | 22.363 | 55.754 | 56.892 |
| C |  | 43.351 | 34.741 | 49.772 | 50.947 |
| D |  | 33.330 | 18.844 | 50.092 | 52.563 |
| E |  | 34.768 | 20.812 | 53.715 | 55.019 |
| F |  | 41.425 | 33.919 | 49.472 | 50.512 |
| G |  | 38.394 | 22.706 | 53.095 | 57.608 |
| H |  | 36.650 | 27.029 | 47.131 | 48.782 |
| I |  | 45.360 | 40.057 | 51.391 | 51.634 |
| K |  | 35.389 | 22.551 | 49.246 | 51.034 |
| L |  | 38.861 | 28.698 | 52.479 | 53.240 |
| M |  | 37.565 | 26.963 | 50.351 | 51.710 |
| N |  | 34.662 | 20.456 | 50.533 | 53.597 |
| P |  | 38.208 | 19.242 | 55.125 | 59.565 |
| Q |  | 35.402 | 22.336 | 51.202 | 52.850 |
| R |  | 35.792 | 24.428 | 49.452 | 51.032 |
| S |  | 35.645 | 24.666 | 46.643 | 49.233 |
| T |  | 39.240 | 30.590 | 47.538 | 49.187 |
| V |  | 48.107 | 43.885 | 52.355 | 51.796 |
| W |  | 38.501 | 31.185 | 47.875 | 47.896 |
| Y |  | 40.024 | 33.443 | 47.949 | 48.181 |
|  |  |  |  |  |  |

M) Standard deviation of secondary structure prediction for Chou-Fasman method using Five-fold cross-validation, with the consideration of actual two-state RSA information.

|  |  | Thresholds | | | | | |
| --- | --- | --- | --- | --- | --- | --- | --- |
|  |  | 4 | 9 | 16 | 25 | 36 | 50 |
| Total |  | 1.114 | 0.760 | 0.414 | 0.606 | 0.644 | 0.547 |
|  |  |  |  |  |  |  |  |
| A |  | 1.173 | 0.511 | 0.187 | 0.450 | 0.546 | 0.549 |
| C |  | 1.541 | 0.762 | 0.556 | 0.595 | 0.198 | 0.615 |
| D |  | 1.295 | 0.866 | 0.385 | 0.403 | 0.313 | 0.552 |
| E |  | 1.201 | 0.403 | 0.184 | 0.387 | 0.383 | 0.686 |
| F |  | 0.874 | 0.511 | 0.664 | 0.519 | 0.332 | 0.462 |
| G |  | 1.254 | 1.060 | 0.488 | 0.754 | 0.903 | 0.643 |
| H |  | 1.004 | 0.588 | 0.324 | 0.631 | 0.257 | 0.563 |
| I |  | 0.922 | 0.346 | 0.208 | 0.350 | 0.503 | 0.381 |
| K |  | 1.233 | 0.468 | 0.366 | 0.398 | 0.364 | 0.472 |
| L |  | 1.217 | 0.398 | 0.263 | 0.373 | 0.386 | 0.286 |
| M |  | 1.259 | 0.450 | 0.364 | 0.386 | 0.305 | 0.330 |
| N |  | 1.238 | 0.951 | 0.452 | 0.547 | 0.402 | 0.543 |
| P |  | 1.543 | 1.014 | 0.265 | 0.393 | 0.233 | 0.661 |
| Q |  | 1.135 | 0.524 | 0.190 | 0.442 | 0.472 | 0.678 |
| R |  | 1.094 | 0.487 | 0.210 | 0.496 | 0.509 | 0.350 |
| S |  | 1.144 | 0.819 | 0.434 | 0.627 | 0.400 | 0.894 |
| T |  | 0.863 | 0.524 | 1.099 | 0.465 | 0.244 | 0.633 |
| V |  | 0.803 | 0.272 | 0.312 | 0.360 | 0.438 | 0.319 |
| W |  | 0.778 | 0.542 | 0.394 | 0.519 | 0.640 | 0.307 |
| Y |  | 0.723 | 0.352 | 0.258 | 0.528 | 0.551 | 0.436 |
|  |  |  |  |  |  |  |  |

N) Standard deviation of secondary structure prediction for Chou-Fasman method using Five-fold cross-validation, with the consideration of predicted two-state RSA information.

|  |  | Thresholds | | | | | |
| --- | --- | --- | --- | --- | --- | --- | --- |
|  |  | 4 | 9 | 16 | 25 | 36 | 50 |
| Total |  | 2.190 | 0.640 | 0.882 | 0.985 | 0.995 | 0.986 |
|  |  |  |  |  |  |  |  |
| A |  | 2.418 | 0.854 | 0.970 | 1.045 | 1.027 | 0.907 |
| C |  | 1.727 | 0.364 | 0.560 | 0.522 | 0.718 | 0.542 |
| D |  | 2.254 | 0.614 | 0.854 | 1.012 | 0.983 | 0.897 |
| E |  | 2.251 | 0.897 | 0.967 | 0.926 | 1.044 | 0.939 |
| F |  | 1.643 | 0.487 | 0.684 | 0.611 | 0.727 | 0.570 |
| G |  | 1.923 | 0.460 | 0.723 | 0.858 | 1.144 | 0.981 |
| H |  | 1.885 | 0.535 | 0.755 | 0.682 | 0.860 | 0.693 |
| I |  | 1.403 | 0.470 | 0.567 | 0.453 | 0.729 | 0.537 |
| K |  | 1.984 | 0.735 | 0.819 | 0.906 | 1.035 | 0.874 |
| L |  | 1.966 | 0.732 | 0.801 | 0.778 | 0.909 | 0.793 |
| M |  | 2.087 | 0.611 | 0.816 | 0.813 | 0.845 | 0.869 |
| N |  | 2.131 | 0.519 | 0.795 | 1.024 | 0.937 | 0.948 |
| P |  | 2.365 | 0.550 | 0.837 | 1.042 | 0.970 | 1.066 |
| Q |  | 2.324 | 0.743 | 0.877 | 0.950 | 1.021 | 1.013 |
| R |  | 2.084 | 0.679 | 0.830 | 0.753 | 0.980 | 0.942 |
| S |  | 1.925 | 0.565 | 0.718 | 0.820 | 0.851 | 0.945 |
| T |  | 1.710 | 0.381 | 0.988 | 0.696 | 0.787 | 0.715 |
| V |  | 1.192 | 0.354 | 0.500 | 0.345 | 0.524 | 0.656 |
| W |  | 1.674 | 0.602 | 0.604 | 0.526 | 0.902 | 0.692 |
| Y |  | 1.535 | 0.458 | 0.541 | 0.731 | 0.770 | 0.561 |
|  |  |  |  |  |  |  |  |

O) Standard deviation of secondary structure prediction for Chou-Fasman method using Five-fold cross-validation, with the consideration of actual three-state RSA information.

|  |  | Thresholds | | | |
| --- | --- | --- | --- | --- | --- |
|  |  | [4,16] | [9,16] | [9,36] | [16,36] |
| Total |  | 0.470 | 0.451 | 0.541 | 0.346 |
|  |  |  |  |  |  |
| A |  | 0.814 | 0.376 | 0.357 | 0.300 |
| C |  | 1.203 | 0.630 | 0.229 | 0.722 |
| D |  | 0.916 | 0.734 | 0.614 | 0.726 |
| E |  | 0.503 | 0.338 | 0.437 | 0.262 |
| F |  | 0.762 | 0.480 | 0.404 | 0.397 |
| G |  | 1.231 | 0.930 | 0.621 | 0.856 |
| H |  | 1.011 | 0.469 | 0.552 | 0.245 |
| I |  | 0.245 | 0.170 | 0.455 | 0.276 |
| K |  | 0.704 | 0.398 | 0.536 | 0.285 |
| L |  | 0.484 | 0.386 | 0.272 | 0.241 |
| M |  | 0.563 | 0.598 | 0.384 | 0.417 |
| N |  | 0.923 | 0.748 | 0.589 | 0.783 |
| P |  | 1.265 | 0.834 | 0.619 | 0.843 |
| Q |  | 0.600 | 0.459 | 0.361 | 0.289 |
| R |  | 0.361 | 0.305 | 0.463 | 0.197 |
| S |  | 1.348 | 0.691 | 0.484 | 0.642 |
| T |  | 1.027 | 0.466 | 0.576 | 0.484 |
| V |  | 0.320 | 0.305 | 0.235 | 0.220 |
| W |  | 0.322 | 0.230 | 0.190 | 0.374 |
| Y |  | 0.205 | 0.332 | 0.471 | 0.260 |
|  |  |  |  |  |  |

P) Standard deviation of secondary structure prediction for Chou-Fasman method using Five-fold cross-validation, with the consideration of predicted three-state RSA information.

|  |  | Thresholds | | | |
| --- | --- | --- | --- | --- | --- |
|  |  | [4,16] | [9,16] | [9,36] | [16,36] |
| Total |  | 1.016 | 0.880 | 0.995 | 0.966 |
|  |  |  |  |  |  |
| A |  | 1.301 | 0.986 | 1.029 | 0.899 |
| C |  | 0.489 | 0.641 | 0.827 | 0.454 |
| D |  | 1.101 | 0.768 | 1.044 | 0.660 |
| E |  | 1.387 | 0.987 | 1.172 | 0.909 |
| F |  | 0.840 | 0.641 | 0.791 | 0.578 |
| G |  | 1.200 | 0.579 | 0.910 | 0.579 |
| H |  | 1.038 | 0.750 | 1.113 | 0.824 |
| I |  | 0.924 | 0.615 | 0.800 | 0.557 |
| K |  | 1.458 | 0.920 | 1.058 | 0.747 |
| L |  | 1.122 | 0.895 | 0.975 | 0.769 |
| M |  | 1.161 | 0.778 | 1.080 | 0.692 |
| N |  | 1.199 | 0.728 | 0.956 | 0.670 |
| P |  | 0.798 | 0.628 | 1.073 | 0.606 |
| Q |  | 1.445 | 0.988 | 1.019 | 0.821 |
| R |  | 1.282 | 0.870 | 1.042 | 0.720 |
| S |  | 0.784 | 0.636 | 0.876 | 0.621 |
| T |  | 0.997 | 0.584 | 0.898 | 0.524 |
| V |  | 0.832 | 0.564 | 0.694 | 0.474 |
| W |  | 1.121 | 0.640 | 0.980 | 0.755 |
| Y |  | 0.973 | 0.556 | 0.975 | 0.600 |
|  |  |  |  |  |  |

Q) Standard deviation of secondary structure prediction for Chou-Fasman method using Five-fold cross-validation, with the consideration of residue-specific classification of actual RSA information.

|  |  | Thresholds | | | |
| --- | --- | --- | --- | --- | --- |
|  |  | Tertiles | Mean±SD | Mean | Median |
| Total |  | 0.840 | 1.043 | 0.593 | 0.541 |
|  |  |  |  |  |  |
| A |  | 0.810 | 0.983 | 0.489 | 0.415 |
| C |  | 0.857 | 1.251 | 0.291 | 0.154 |
| D |  | 0.578 | 1.132 | 0.584 | 0.571 |
| E |  | 0.686 | 0.965 | 0.400 | 0.299 |
| F |  | 0.962 | 0.756 | 0.552 | 0.534 |
| G |  | 0.672 | 1.906 | 0.553 | 0.641 |
| H |  | 0.397 | 0.300 | 0.317 | 0.346 |
| I |  | 0.605 | 1.194 | 0.212 | 0.186 |
| K |  | 0.618 | 0.735 | 0.366 | 0.329 |
| L |  | 0.772 | 1.231 | 0.316 | 0.223 |
| M |  | 0.864 | 1.157 | 0.395 | 0.377 |
| N |  | 0.938 | 1.452 | 0.580 | 0.563 |
| P |  | 0.670 | 1.657 | 0.584 | 0.673 |
| Q |  | 0.543 | 0.281 | 0.474 | 0.380 |
| R |  | 0.830 | 0.411 | 0.281 | 0.302 |
| S |  | 0.689 | 0.943 | 0.420 | 0.362 |
| T |  | 0.552 | 1.443 | 0.247 | 0.247 |
| V |  | 0.484 | 1.024 | 0.198 | 0.282 |
| W |  | 0.581 | 0.512 | 0.497 | 0.515 |
| Y |  | 0.231 | 0.739 | 0.493 | 0.529 |
|  |  |  |  |  |  |

R) Standard deviation of secondary structure prediction for Chou-Fasman method using Five-fold cross-validation, with the consideration of residue-specific classification of predicted RSA information.

|  |  | Thresholds | | | |
| --- | --- | --- | --- | --- | --- |
|  |  | Tertiles | Mean±SD | Mean | Median |
| Total |  | 0.748 | 0.519 | 0.642 | 0.325 |
|  |  |  |  |  |  |
| A |  | 0.523 | 0.269 | 0.422 | 0.308 |
| C |  | 0.690 | 0.227 | 0.575 | 0.216 |
| D |  | 0.678 | 0.332 | 0.226 | 0.286 |
| E |  | 0.627 | 0.210 | 0.486 | 0.288 |
| F |  | 0.396 | 0.221 | 0.570 | 0.197 |
| G |  | 0.765 | 0.363 | 1.153 | 0.268 |
| H |  | 0.307 | 0.296 | 0.386 | 0.263 |
| I |  | 0.348 | 0.333 | 0.325 | 0.191 |
| K |  | 0.717 | 0.282 | 0.347 | 0.260 |
| L |  | 0.202 | 0.254 | 0.346 | 0.276 |
| M |  | 0.443 | 0.403 | 0.711 | 0.278 |
| N |  | 0.739 | 0.405 | 0.300 | 0.278 |
| P |  | 0.662 | 0.279 | 0.779 | 0.291 |
| Q |  | 0.443 | 0.255 | 0.344 | 0.255 |
| R |  | 0.478 | 0.198 | 0.349 | 0.323 |
| S |  | 0.626 | 0.324 | 0.908 | 0.214 |
| T |  | 0.379 | 0.204 | 0.198 | 0.230 |
| V |  | 0.185 | 0.210 | 0.213 | 0.173 |
| W |  | 0.259 | 0.379 | 0.358 | 0.415 |
| Y |  | 0.607 | 0.213 | 0.320 | 0.253 |
|  |  |  |  |  |  |
