# Supplementary material for: Impact of residue accessible surface area on the prediction of protein secondary structures
Source: BMC Bioinformatics. 2008 Aug 31;9:357. doi: 10.1186/1471-2105-9-357 (PMC2553345; doi:10.1186/1471-2105-9-357)
Supplement: Additional file 4 — Accuracy of secondary structure prediction for HMM method, with the consideration of actual and predicted RSA information. [file 1471-2105-9-357-S4.doc]

A) Accuracy of secondary structure prediction for HMM method using leave-one-out cross-validation, with the consideration of actual two-state RSA information. Totally, 1571044 residues were present in the assessed dataset. For each of the twenty amino acids the accuracy of prediction is reported separately.

|  |  | Thresholds | | | | | |
| --- | --- | --- | --- | --- | --- | --- | --- |
|  |  | 4 | 9 | 16 | 25 | 36 | 50 |
| Correct |  | 755311 | 747781 | 743349 | 739430 | 737668 | 737764 |
| False |  | 815733 | 823263 | 827695 | 831614 | 833376 | 833280 |
| Q3 |  | 49.885 | 49.433 | 49.112 | 48.862 | 48.670 | 48.548 |
| SD |  | 14.448 | 14.556 | 14.557 | 14.601 | 14.765 | 15.334 |
|  |  |  |  |  |  |  |  |
| A |  | 49.034 | 48.522 | 48.452 | 48.380 | 48.817 | 50.135 |
| C |  | 46.436 | 45.808 | 45.494 | 45.217 | 44.894 | 44.436 |
| D |  | 51.495 | 51.195 | 50.919 | 50.399 | 49.771 | 48.643 |
| E |  | 50.097 | 49.683 | 49.596 | 49.501 | 49.885 | 50.919 |
| F |  | 43.404 | 42.940 | 42.681 | 42.428 | 42.503 | 42.588 |
| G |  | 56.226 | 55.659 | 54.871 | 53.715 | 52.296 | 50.470 |
| H |  | 47.269 | 46.997 | 46.817 | 46.467 | 46.364 | 46.488 |
| I |  | 41.018 | 40.338 | 40.041 | 40.168 | 40.447 | 40.756 |
| K |  | 48.704 | 48.327 | 48.132 | 48.189 | 48.291 | 48.496 |
| L |  | 45.554 | 44.893 | 44.695 | 44.814 | 45.145 | 45.998 |
| M |  | 46.675 | 46.103 | 46.006 | 45.853 | 46.359 | 47.541 |
| N |  | 52.457 | 52.201 | 51.719 | 51.200 | 50.399 | 49.348 |
| P |  | 58.581 | 58.235 | 57.601 | 56.545 | 55.564 | 53.712 |
| Q |  | 49.208 | 48.652 | 48.585 | 48.342 | 48.571 | 49.495 |
| R |  | 47.624 | 46.998 | 46.885 | 46.746 | 46.921 | 47.535 |
| S |  | 49.132 | 48.832 | 48.445 | 47.959 | 47.608 | 47.072 |
| T |  | 46.531 | 46.114 | 45.726 | 45.453 | 45.020 | 44.689 |
| V |  | 39.999 | 39.406 | 39.107 | 39.246 | 39.364 | 39.479 |
| W |  | 43.046 | 42.526 | 42.467 | 42.462 | 42.715 | 43.018 |
| Y |  | 42.915 | 42.552 | 42.455 | 42.581 | 42.714 | 42.721 |
|  |  |  |  |  |  |  |  |

B) Accuracy of secondary structure prediction for HMM method using leave-one-out cross-validation, with the consideration of predicted two-state RSA information.

|  |  | Thresholds | | | | | |
| --- | --- | --- | --- | --- | --- | --- | --- |
|  |  | 4 | 9 | 16 | 25 | 36 | 50 |
| Correct |  | 741770 | 741427 | 720676 | 703415 | 698549 | 684931 |
| False |  | 829274 | 829617 | 850368 | 867629 | 872495 | 886113 |
| Q3 |  | 49.913 | 49.167 | 47.862 | 45.772 | 45.459 | 44.242 |
| SD |  | 13.823 | 14.627 | 13.965 | 15.798 | 15.764 | 16.946 |
|  |  |  |  |  |  |  |  |
| A |  | 44.112 | 47.703 | 43.048 | 48.167 | 49.049 | 49.080 |
| C |  | 46.543 | 45.323 | 43.568 | 42.127 | 42.094 | 40.649 |
| D |  | 53.623 | 51.270 | 53.670 | 46.292 | 44.596 | 44.463 |
| E |  | 45.997 | 49.177 | 45.459 | 50.025 | 50.364 | 49.607 |
| F |  | 41.599 | 42.295 | 38.838 | 41.273 | 41.725 | 40.070 |
| G |  | 61.136 | 54.874 | 60.878 | 43.884 | 40.812 | 40.819 |
| H |  | 47.713 | 47.039 | 46.716 | 43.533 | 42.726 | 42.930 |
| I |  | 37.978 | 39.542 | 34.295 | 40.127 | 41.192 | 38.585 |
| K |  | 47.327 | 48.265 | 46.696 | 47.087 | 46.910 | 46.047 |
| L |  | 41.467 | 44.224 | 39.426 | 45.070 | 45.865 | 45.028 |
| M |  | 42.630 | 45.451 | 41.555 | 45.832 | 46.792 | 46.210 |
| N |  | 55.628 | 52.322 | 55.690 | 46.478 | 44.187 | 44.095 |
| P |  | 63.360 | 58.317 | 64.061 | 48.526 | 45.649 | 45.143 |
| Q |  | 46.360 | 48.189 | 45.594 | 47.975 | 48.150 | 47.692 |
| R |  | 45.443 | 46.673 | 44.586 | 46.074 | 46.264 | 45.617 |
| S |  | 50.763 | 48.819 | 50.386 | 43.911 | 42.849 | 42.938 |
| T |  | 47.877 | 45.977 | 46.148 | 42.106 | 41.400 | 40.450 |
| V |  | 38.138 | 38.766 | 33.779 | 38.743 | 39.745 | 36.406 |
| W |  | 40.996 | 42.090 | 39.241 | 41.584 | 42.398 | 41.051 |
| Y |  | 41.583 | 42.331 | 39.441 | 41.430 | 41.866 | 40.176 |
|  |  |  |  |  |  |  |  |

C) Accuracy of secondary structure prediction for HMM method using leave-one-out cross-validation, with the consideration of actual three-state RSA information.

|  |  | Thresholds | | | |
| --- | --- | --- | --- | --- | --- |
|  |  | [4,16] | [9,16] | [9,36] | [16,36] |
| Correct |  | 744001 | 740349 | 740517 | 740517 |
| False |  | 827043 | 830695 | 830527 | 830527 |
| Q3 |  | 49.710 | 49.271 | 49.563 | 49.563 |
| SD |  | 14.150 | 14.397 | 13.970 | 13.970 |
|  |  |  |  |  |  |
| A |  | 45.607 | 46.130 | 45.041 | 45.041 |
| C |  | 46.228 | 45.660 | 46.099 | 46.099 |
| D |  | 52.867 | 52.198 | 52.658 | 52.658 |
| E |  | 47.285 | 47.765 | 46.872 | 46.872 |
| F |  | 42.047 | 42.133 | 41.794 | 41.794 |
| G |  | 59.551 | 58.031 | 59.210 | 59.210 |
| H |  | 47.410 | 47.091 | 47.518 | 47.518 |
| I |  | 38.503 | 38.696 | 38.341 | 38.341 |
| K |  | 47.559 | 47.692 | 47.583 | 47.583 |
| L |  | 42.567 | 42.884 | 42.097 | 42.097 |
| M |  | 43.805 | 44.172 | 43.458 | 43.458 |
| N |  | 54.671 | 53.646 | 54.601 | 54.601 |
| P |  | 61.966 | 60.403 | 61.772 | 61.772 |
| Q |  | 47.243 | 47.361 | 46.859 | 46.859 |
| R |  | 46.087 | 45.984 | 45.733 | 45.733 |
| S |  | 50.248 | 49.616 | 50.266 | 50.266 |
| T |  | 47.203 | 46.488 | 47.187 | 47.187 |
| V |  | 38.298 | 38.193 | 38.330 | 38.330 |
| W |  | 41.690 | 41.584 | 41.276 | 41.276 |
| Y |  | 41.764 | 41.858 | 41.904 | 41.904 |
|  |  |  |  |  |  |

D) Accuracy of secondary structure prediction for HMM method using leave-one-out cross-validation, with the consideration of predicted three-state RSA information.

|  |  | Thresholds | | | |
| --- | --- | --- | --- | --- | --- |
|  |  | [4,16] | [9,16] | [9,36] | [16,36] |
| Correct |  | 746675 | 745279 | 732413 | 724377 |
| False |  | 824369 | 825765 | 838631 | 846667 |
| Q3 |  | 49.505 | 49.240 | 48.653 | 47.903 |
| SD |  | 13.086 | 14.451 | 14.265 | 14.722 |
|  |  |  |  |  |  |
| A |  | 45.277 | 47.588 | 45.537 | 45.550 |
| C |  | 44.455 | 44.039 | 43.480 | 42.995 |
| D |  | 54.373 | 52.105 | 51.708 | 50.864 |
| E |  | 47.660 | 49.160 | 47.577 | 47.591 |
| F |  | 40.199 | 41.831 | 41.315 | 41.033 |
| G |  | 62.309 | 57.831 | 56.732 | 55.026 |
| H |  | 47.619 | 47.335 | 46.652 | 46.047 |
| I |  | 37.270 | 38.572 | 38.003 | 37.705 |
| K |  | 47.765 | 48.425 | 47.726 | 47.459 |
| L |  | 41.787 | 43.907 | 42.470 | 42.432 |
| M |  | 44.540 | 45.375 | 43.808 | 43.652 |
| N |  | 56.176 | 53.352 | 53.231 | 52.221 |
| P |  | 64.265 | 59.286 | 59.442 | 57.723 |
| Q |  | 47.491 | 48.307 | 46.980 | 46.899 |
| R |  | 46.356 | 46.938 | 45.826 | 45.687 |
| S |  | 51.182 | 49.542 | 49.305 | 48.464 |
| T |  | 47.052 | 46.359 | 46.090 | 45.388 |
| V |  | 36.434 | 37.864 | 37.890 | 37.427 |
| W |  | 40.904 | 42.058 | 41.240 | 41.033 |
| Y |  | 41.625 | 41.961 | 41.587 | 41.357 |
|  |  |  |  |  |  |

E) Accuracy of secondary structure prediction for HMM method using leave-one-out cross-validation, with the consideration of residue-specific classification of actual RSA information.

|  |  | Thresholds | | | |
| --- | --- | --- | --- | --- | --- |
|  |  | Tertiles | Mean±SD | Mean | Median |
| Correct |  | 767390 | 756644 | 750237 | 743391 |
| False |  | 803654 | 814400 | 820807 | 827653 |
| Q3 |  | 50.284 | 49.584 | 49.893 | 49.337 |
| SD |  | 12.755 | 14.586 | 14.158 | 14.369 |
|  |  |  |  |  |  |
| A |  | 48.614 | 50.231 | 47.509 | 47.631 |
| C |  | 45.402 | 44.487 | 46.487 | 45.813 |
| D |  | 54.228 | 51.302 | 51.945 | 51.102 |
| E |  | 50.583 | 51.515 | 48.796 | 49.006 |
| F |  | 41.277 | 42.811 | 42.993 | 42.558 |
| G |  | 61.262 | 55.913 | 57.027 | 55.566 |
| H |  | 48.495 | 47.781 | 47.298 | 46.845 |
| I |  | 39.593 | 40.218 | 40.414 | 40.087 |
| K |  | 49.176 | 49.345 | 48.355 | 48.263 |
| L |  | 44.489 | 46.053 | 44.357 | 44.310 |
| M |  | 47.496 | 47.271 | 45.299 | 45.316 |
| N |  | 55.811 | 52.148 | 53.217 | 52.292 |
| P |  | 63.674 | 57.395 | 59.904 | 58.311 |
| Q |  | 49.862 | 49.936 | 48.064 | 48.044 |
| R |  | 48.148 | 48.242 | 46.764 | 46.660 |
| S |  | 51.228 | 49.105 | 49.609 | 48.892 |
| T |  | 47.308 | 46.172 | 46.976 | 46.095 |
| V |  | 38.072 | 39.161 | 39.634 | 39.336 |
| W |  | 42.623 | 43.560 | 42.283 | 42.343 |
| Y |  | 42.705 | 42.811 | 42.603 | 42.433 |
|  |  |  |  |  |  |

F) Accuracy of secondary structure prediction for HMM method using leave-one-out cross-validation, with the consideration of residue-specific classification of predicted RSA information.

|  |  | Thresholds | | | |
| --- | --- | --- | --- | --- | --- |
|  |  | Tertiles | Mean±SD | Mean | Median |
| Correct |  | 745330 | 723802 | 746312 | 723590 |
| False |  | 825714 | 847242 | 824732 | 847454 |
| Q3 |  | 48.728 | 47.231 | 49.360 | 47.674 |
| SD |  | 15.056 | 15.621 | 12.699 | 14.553 |
|  |  |  |  |  |  |
| A |  | 50.274 | 49.086 | 45.788 | 46.532 |
| C |  | 44.021 | 43.318 | 44.154 | 43.244 |
| D |  | 50.067 | 48.190 | 54.117 | 49.279 |
| E |  | 51.109 | 50.502 | 47.488 | 48.809 |
| F |  | 42.484 | 41.734 | 40.405 | 42.156 |
| G |  | 53.694 | 48.782 | 61.097 | 50.543 |
| H |  | 46.948 | 45.178 | 47.492 | 45.129 |
| I |  | 39.851 | 39.750 | 37.669 | 40.183 |
| K |  | 48.903 | 48.091 | 47.473 | 47.643 |
| L |  | 45.932 | 45.458 | 41.886 | 43.996 |
| M |  | 46.928 | 46.435 | 44.768 | 44.370 |
| N |  | 50.835 | 48.783 | 55.964 | 50.031 |
| P |  | 55.144 | 52.177 | 64.187 | 54.995 |
| Q |  | 49.680 | 48.676 | 47.437 | 47.153 |
| R |  | 47.845 | 47.084 | 46.392 | 45.917 |
| S |  | 48.078 | 46.252 | 50.961 | 46.679 |
| T |  | 45.164 | 43.492 | 46.974 | 44.694 |
| V |  | 38.634 | 38.299 | 37.000 | 39.959 |
| W |  | 43.073 | 42.081 | 41.566 | 41.639 |
| Y |  | 42.709 | 41.982 | 41.972 | 42.147 |
|  |  |  |  |  |  |

G) Accuracy of secondary structure prediction for HMM method using Five-fold cross-validation, with the consideration of actual two-state RSA information.

|  |  | Thresholds | | | | | |
| --- | --- | --- | --- | --- | --- | --- | --- |
|  |  | 4 | 9 | 16 | 25 | 36 | 50 |
| Correct |  | 737161.450 | 730456.566 | 726646.534 | 723092.078 | 721436.370 | 721712.756 |
| False |  | 833882.550 | 840587.434 | 844397.466 | 847951.922 | 849607.630 | 849331.244 |
| Q3 |  | 46.922 | 46.495 | 46.252 | 46.026 | 45.921 | 45.938 |
|  |  |  |  |  |  |  |  |
| A |  | 49.389 | 48.933 | 48.870 | 48.812 | 49.194 | 50.374 |
| C |  | 44.729 | 44.176 | 43.884 | 43.641 | 43.348 | 42.965 |
| D |  | 49.424 | 49.169 | 48.928 | 48.447 | 47.906 | 46.912 |
| E |  | 50.271 | 49.906 | 49.826 | 49.745 | 50.084 | 51.005 |
| F |  | 42.723 | 42.302 | 42.084 | 41.850 | 41.919 | 41.995 |
| G |  | 52.036 | 51.574 | 50.857 | 49.787 | 48.593 | 46.927 |
| H |  | 45.958 | 45.728 | 45.569 | 45.263 | 45.150 | 45.263 |
| I |  | 40.626 | 40.028 | 39.758 | 39.871 | 40.123 | 40.394 |
| K |  | 48.108 | 47.771 | 47.597 | 47.658 | 47.746 | 47.924 |
| L |  | 45.820 | 45.228 | 45.054 | 45.157 | 45.455 | 46.217 |
| M |  | 46.454 | 45.947 | 45.861 | 45.724 | 46.174 | 47.228 |
| N |  | 49.840 | 49.650 | 49.176 | 48.735 | 47.992 | 47.109 |
| P |  | 54.423 | 54.166 | 53.596 | 52.678 | 51.719 | 50.122 |
| Q |  | 49.024 | 48.529 | 48.466 | 48.250 | 48.454 | 49.279 |
| R |  | 47.275 | 46.720 | 46.615 | 46.493 | 46.652 | 47.198 |
| S |  | 47.130 | 46.899 | 46.529 | 46.099 | 45.815 | 45.332 |
| T |  | 44.578 | 44.206 | 43.843 | 43.613 | 43.204 | 42.919 |
| V |  | 39.108 | 38.575 | 38.316 | 38.452 | 38.557 | 38.662 |
| W |  | 42.714 | 42.253 | 42.205 | 42.197 | 42.421 | 42.694 |
| Y |  | 42.177 | 41.855 | 41.774 | 41.892 | 42.000 | 42.011 |
|  |  |  |  |  |  |  |  |

H) Accuracy of secondary structure prediction for HMM method using Five-fold cross-validation, with the consideration of predicted two-state RSA information.

|  |  | Thresholds | | | | | |
| --- | --- | --- | --- | --- | --- | --- | --- |
|  |  | 4 | 9 | 16 | 25 | 36 | 50 |
| Correct |  | 724762.728 | 724449.971 | 706259.537 | 690826.376 | 686406.176 | 674176.423 |
| False |  | 846281.272 | 846594.029 | 864784.463 | 880217.624 | 884637.824 | 896867.577 |
| Q3 |  | 46.133 | 46.113 | 44.955 | 43.972 | 43.691 | 42.913 |
|  |  |  |  |  |  |  |  |
| A |  | 45.001 | 48.189 | 44.044 | 48.601 | 49.386 | 49.415 |
| C |  | 44.815 | 43.739 | 42.170 | 40.876 | 40.844 | 39.553 |
| D |  | 51.338 | 49.233 | 51.351 | 44.805 | 43.268 | 43.163 |
| E |  | 46.599 | 49.433 | 46.126 | 50.190 | 50.490 | 49.816 |
| F |  | 41.096 | 41.724 | 38.638 | 40.811 | 41.217 | 39.736 |
| G |  | 56.430 | 50.869 | 56.145 | 41.076 | 38.323 | 38.289 |
| H |  | 46.343 | 45.740 | 45.463 | 42.619 | 41.906 | 42.071 |
| I |  | 37.915 | 39.307 | 34.634 | 39.830 | 40.776 | 38.454 |
| K |  | 46.862 | 47.692 | 46.300 | 46.652 | 46.490 | 45.722 |
| L |  | 42.173 | 44.621 | 40.352 | 45.367 | 46.076 | 45.331 |
| M |  | 42.843 | 45.353 | 41.884 | 45.695 | 46.551 | 46.027 |
| N |  | 52.636 | 49.701 | 52.739 | 44.497 | 42.448 | 42.361 |
| P |  | 58.700 | 54.164 | 59.291 | 45.436 | 42.902 | 42.419 |
| Q |  | 46.469 | 48.092 | 45.782 | 47.903 | 48.059 | 47.651 |
| R |  | 45.317 | 46.411 | 44.551 | 45.878 | 46.044 | 45.467 |
| S |  | 48.575 | 46.873 | 48.247 | 42.492 | 41.543 | 41.637 |
| T |  | 45.759 | 44.071 | 44.230 | 40.610 | 39.989 | 39.136 |
| V |  | 37.447 | 38.000 | 33.564 | 37.975 | 38.872 | 35.900 |
| W |  | 40.881 | 41.849 | 39.316 | 41.403 | 42.125 | 40.926 |
| Y |  | 40.984 | 41.654 | 39.083 | 40.851 | 41.237 | 39.738 |
|  |  |  |  |  |  |  |  |

I) Accuracy of secondary structure prediction for HMM method using Five-fold cross-validation, with the consideration of actual three-state RSA information.

|  |  | Thresholds | | | |
| --- | --- | --- | --- | --- | --- |
|  |  | [4,16] | [9,16] | [9,36] | [16,36] |
| Correct |  | 726907.890 | 724054.312 | 723900.200 | 724071.805 |
| False |  | 844136.110 | 846989.688 | 847143.800 | 846972.195 |
| Q3 |  | 46.269 | 46.087 | 46.078 | 46.089 |
|  |  |  |  |  |  |
| A |  | 46.330 | 46.803 | 45.826 | 45.833 |
| C |  | 44.572 | 44.064 | 44.437 | 44.427 |
| D |  | 50.687 | 50.063 | 50.469 | 50.498 |
| E |  | 47.765 | 48.188 | 47.402 | 47.393 |
| F |  | 41.506 | 41.589 | 41.284 | 41.289 |
| G |  | 55.048 | 53.705 | 54.763 | 54.736 |
| H |  | 46.101 | 45.826 | 46.203 | 46.202 |
| I |  | 38.391 | 38.560 | 38.245 | 38.245 |
| K |  | 47.095 | 47.202 | 47.109 | 47.107 |
| L |  | 43.163 | 43.435 | 42.734 | 42.739 |
| M |  | 43.899 | 44.225 | 43.591 | 43.592 |
| N |  | 51.809 | 50.918 | 51.766 | 51.789 |
| P |  | 57.494 | 56.005 | 57.335 | 57.279 |
| Q |  | 47.271 | 47.376 | 46.927 | 46.926 |
| R |  | 45.905 | 45.814 | 45.586 | 45.587 |
| S |  | 48.159 | 47.562 | 48.167 | 48.146 |
| T |  | 45.190 | 44.530 | 45.164 | 45.169 |
| V |  | 37.592 | 37.513 | 37.632 | 37.630 |
| W |  | 41.510 | 41.411 | 41.136 | 41.139 |
| Y |  | 41.161 | 41.240 | 41.292 | 41.285 |
|  |  |  |  |  |  |

J) Accuracy of secondary structure prediction for HMM method using Five-fold cross-validation, with the consideration of predicted three-state RSA information.

|  |  | Thresholds | | | |
| --- | --- | --- | --- | --- | --- |
|  |  | [4,16] | [9,16] | [9,36] | [16,36] |
| Correct |  | 729323.836 | 728381.768 | 716568.378 | 709550.486 |
| False |  | 841720.164 | 842662.232 | 854475.622 | 861493.514 |
| Q3 |  | 46.423 | 46.363 | 45.611 | 45.164 |
|  |  |  |  |  |  |
| A |  | 46.040 | 48.096 | 46.263 | 46.286 |
| C |  | 42.983 | 42.602 | 42.105 | 41.675 |
| D |  | 51.989 | 49.985 | 49.647 | 48.894 |
| E |  | 48.094 | 49.431 | 48.028 | 48.036 |
| F |  | 39.862 | 41.311 | 40.859 | 40.606 |
| G |  | 57.498 | 53.447 | 52.496 | 50.926 |
| H |  | 46.270 | 46.021 | 45.420 | 44.875 |
| I |  | 37.288 | 38.449 | 37.943 | 37.678 |
| K |  | 47.261 | 47.853 | 47.230 | 46.997 |
| L |  | 42.459 | 44.341 | 43.062 | 43.028 |
| M |  | 44.549 | 45.295 | 43.902 | 43.764 |
| N |  | 53.164 | 50.654 | 50.542 | 49.609 |
| P |  | 59.537 | 55.086 | 55.163 | 53.671 |
| Q |  | 47.484 | 48.215 | 47.034 | 46.963 |
| R |  | 46.135 | 46.663 | 45.670 | 45.546 |
| S |  | 48.978 | 47.504 | 47.299 | 46.579 |
| T |  | 45.028 | 44.427 | 44.197 | 43.570 |
| V |  | 35.933 | 37.215 | 37.236 | 36.825 |
| W |  | 40.802 | 41.830 | 41.104 | 40.921 |
| Y |  | 41.027 | 41.335 | 40.994 | 40.804 |
|  |  |  |  |  |  |

K) Accuracy of secondary structure prediction for HMM method using Five-fold cross-validation, with the consideration of residue-specific classification of actual RSA information.

|  |  | Thresholds | | | |
| --- | --- | --- | --- | --- | --- |
|  |  | Tertiles | Mean±SD | Mean | Median |
| Correct |  | 747820.371 | 740152.980 | 728576.990 | 734641.866 |
| False |  | 823223.629 | 830891.020 | 842467.010 | 836402.134 |
| Q3 |  | 47.600 | 47.112 | 46.375 | 46.761 |
|  |  |  |  |  |  |
| A |  | 49.010 | 50.623 | 48.310 | 48.200 |
| C |  | 43.795 | 43.089 | 44.289 | 44.890 |
| D |  | 51.862 | 49.373 | 49.188 | 49.968 |
| E |  | 50.699 | 51.703 | 49.475 | 49.279 |
| F |  | 40.822 | 42.315 | 42.085 | 42.479 |
| G |  | 56.578 | 51.818 | 51.471 | 52.833 |
| H |  | 47.072 | 46.541 | 45.696 | 46.103 |
| I |  | 39.360 | 40.039 | 39.925 | 40.216 |
| K |  | 48.526 | 48.823 | 47.854 | 47.942 |
| L |  | 44.867 | 46.417 | 44.869 | 44.911 |
| M |  | 47.183 | 47.133 | 45.392 | 45.376 |
| N |  | 52.788 | 49.642 | 49.785 | 50.593 |
| P |  | 58.919 | 53.454 | 54.260 | 55.709 |
| Q |  | 49.600 | 49.822 | 48.140 | 48.158 |
| R |  | 47.741 | 47.972 | 46.563 | 46.654 |
| S |  | 49.036 | 47.230 | 47.032 | 47.664 |
| T |  | 45.267 | 44.317 | 44.292 | 45.045 |
| V |  | 37.394 | 38.474 | 38.621 | 38.887 |
| W |  | 42.337 | 43.305 | 42.221 | 42.169 |
| Y |  | 41.989 | 42.206 | 41.871 | 42.021 |
|  |  |  |  |  |  |

L) Accuracy of secondary structure prediction for HMM method using Five-fold cross-validation, with the consideration of residue-specific classification of predicted RSA information.

|  |  | Thresholds | | | |
| --- | --- | --- | --- | --- | --- |
|  |  | Tertiles | Mean±SD | Mean | Median |
| Correct |  | 731611.782 | 710227.956 | 710149.031 | 730491.752 |
| False |  | 839432.218 | 860816.044 | 860894.969 | 840552.248 |
| Q3 |  | 46.569 | 45.207 | 45.202 | 46.497 |
|  |  |  |  |  |  |
| A |  | 50.788 | 49.553 | 47.269 | 46.607 |
| C |  | 42.751 | 42.026 | 41.965 | 42.790 |
| D |  | 48.326 | 46.569 | 47.548 | 51.877 |
| E |  | 51.453 | 50.745 | 49.223 | 48.055 |
| F |  | 42.102 | 41.318 | 41.688 | 40.128 |
| G |  | 49.901 | 45.484 | 46.992 | 56.490 |
| H |  | 45.868 | 44.175 | 44.143 | 46.232 |
| I |  | 39.798 | 39.582 | 39.962 | 37.732 |
| K |  | 48.524 | 47.657 | 47.253 | 47.114 |
| L |  | 46.418 | 45.833 | 44.521 | 42.655 |
| M |  | 46.928 | 46.339 | 44.494 | 44.856 |
| N |  | 48.568 | 46.608 | 47.727 | 53.060 |
| P |  | 51.474 | 48.801 | 51.304 | 59.516 |
| Q |  | 49.702 | 48.651 | 47.282 | 47.549 |
| R |  | 47.715 | 46.888 | 45.841 | 46.277 |
| S |  | 46.408 | 44.672 | 45.021 | 48.838 |
| T |  | 43.495 | 41.942 | 42.986 | 45.026 |
| V |  | 38.072 | 37.671 | 39.147 | 36.512 |
| W |  | 42.957 | 41.945 | 41.541 | 41.485 |
| Y |  | 42.194 | 41.439 | 41.581 | 41.429 |
|  |  |  |  |  |  |

M) Standard deviation of secondary structure prediction for HMM method using Five-fold cross-validation, with the consideration of actual two-state RSA information.

|  |  | Thresholds | | | | | |
| --- | --- | --- | --- | --- | --- | --- | --- |
|  |  | 4 | 9 | 16 | 25 | 36 | 50 |
| Total |  | 1.508 | 0.625 | 0.443 | 3.031 | 0.453 | 0.758 |
|  |  |  |  |  |  |  |  |
| A |  | 1.508 | 0.438 | 0.465 | 0.477 | 0.589 | 0.621 |
| C |  | 1.094 | 0.393 | 0.512 | 0.726 | 0.526 | 0.398 |
| D |  | 1.080 | 0.334 | 0.291 | 0.362 | 0.324 | 0.554 |
| E |  | 0.747 | 0.528 | 0.255 | 0.350 | 0.491 | 0.550 |
| F |  | 1.020 | 0.346 | 0.292 | 0.269 | 0.262 | 0.628 |
| G |  | 1.253 | 0.507 | 0.695 | 0.363 | 0.353 | 0.584 |
| H |  | 0.647 | 2.129 | 0.411 | 0.245 | 0.411 | 0.299 |
| I |  | 0.964 | 0.490 | 0.638 | 0.274 | 0.441 | 0.795 |
| K |  | 1.013 | 0.391 | 0.667 | 0.300 | 0.288 | 0.693 |
| L |  | 1.288 | 0.591 | 0.497 | 0.329 | 0.428 | 0.447 |
| M |  | 0.907 | 0.494 | 0.328 | 1.762 | 0.388 | 0.581 |
| N |  | 1.348 | 0.348 | 0.334 | 0.260 | 0.426 | 0.648 |
| P |  | 1.401 | 0.465 | 0.747 | 0.286 | 0.344 | 0.563 |
| Q |  | 1.426 | 0.389 | 0.977 | 0.344 | 0.235 | 0.477 |
| R |  | 1.357 | 0.329 | 0.245 | 0.390 | 0.242 | 0.669 |
| S |  | 0.945 | 0.349 | 0.315 | 0.261 | 0.513 | 0.464 |
| T |  | 0.773 | 0.249 | 0.356 | 0.338 | 0.317 | 0.496 |
| V |  | 1.139 | 0.609 | 0.381 | 0.500 | 0.485 | 0.844 |
| W |  | 1.138 | 0.394 | 0.366 | 0.428 | 0.476 | 0.510 |
| Y |  | 0.110 | 0.034 | 0.016 | 0.017 | 0.026 | 0.056 |
|  |  |  |  |  |  |  |  |

N) Standard deviation of secondary structure prediction for HMM method using Five-fold cross-validation, with the consideration of predicted two-state RSA information.

|  |  | Thresholds | | | | | |
| --- | --- | --- | --- | --- | --- | --- | --- |
|  |  | 4 | 9 | 16 | 25 | 36 | 50 |
| Total |  | 2.554 | 0.798 | 0.541 | 0.517 | 0.982 | 0.618 |
|  |  |  |  |  |  |  |  |
| A |  | 2.454 | 0.570 | 0.468 | 0.499 | 0.439 | 0.571 |
| C |  | 1.916 | 0.377 | 0.471 | 0.419 | 2.927 | 0.375 |
| D |  | 2.122 | 0.419 | 0.470 | 0.317 | 2.442 | 0.327 |
| E |  | 1.553 | 0.328 | 0.473 | 0.306 | 0.332 | 0.401 |
| F |  | 1.881 | 0.437 | 0.327 | 0.493 | 2.021 | 2.628 |
| G |  | 2.297 | 0.649 | 0.733 | 0.765 | 0.523 | 0.413 |
| H |  | 1.491 | 0.978 | 2.641 | 0.727 | 0.242 | 0.625 |
| I |  | 2.373 | 0.664 | 0.559 | 0.387 | 0.236 | 0.618 |
| K |  | 1.875 | 0.373 | 0.317 | 0.255 | 0.296 | 0.582 |
| L |  | 1.755 | 0.401 | 0.405 | 0.245 | 0.401 | 0.506 |
| M |  | 2.104 | 0.460 | 0.364 | 0.686 | 0.272 | 0.299 |
| N |  | 2.303 | 0.593 | 0.440 | 0.266 | 0.549 | 0.375 |
| P |  | 2.363 | 0.577 | 0.638 | 0.305 | 0.297 | 0.526 |
| Q |  | 2.106 | 0.552 | 0.498 | 0.288 | 0.240 | 0.458 |
| R |  | 2.096 | 0.518 | 0.436 | 0.271 | 0.292 | 0.485 |
| S |  | 1.467 | 0.337 | 0.260 | 0.239 | 2.236 | 0.372 |
| T |  | 1.770 | 0.260 | 0.339 | 0.294 | 1.150 | 0.361 |
| V |  | 1.589 | 0.625 | 0.494 | 0.505 | 0.374 | 0.481 |
| W |  | 1.944 | 0.277 | 0.456 | 0.479 | 0.302 | 0.256 |
| Y |  | 0.190 | 0.043 | 0.038 | 0.024 | 0.012 | 0.034 |
|  |  |  |  |  |  |  |  |

O) Standard deviation of secondary structure prediction for HMM method using Five-fold cross-validation, with the consideration of actual three-state RSA information.

|  |  | Thresholds | | | |
| --- | --- | --- | --- | --- | --- |
|  |  | [4,16] | [9,16] | [9,36] | [16,36] |
| Total |  | 1.161 | 0.581 | 0.774 | 0.492 |
|  |  |  |  |  |  |
| A |  | 0.823 | 0.387 | 0.376 | 0.486 |
| C |  | 0.588 | 0.429 | 0.574 | 0.361 |
| D |  | 1.086 | 0.506 | 0.673 | 0.325 |
| E |  | 1.096 | 0.557 | 0.727 | 0.317 |
| F |  | 1.184 | 0.553 | 0.739 | 0.329 |
| G |  | 0.947 | 0.396 | 0.852 | 0.455 |
| H |  | 0.910 | 0.337 | 0.428 | 0.670 |
| I |  | 1.445 | 0.501 | 0.694 | 0.490 |
| K |  | 1.003 | 0.434 | 0.628 | 0.355 |
| L |  | 0.807 | 0.818 | 0.429 | 0.733 |
| M |  | 0.960 | 0.309 | 0.534 | 0.434 |
| N |  | 1.340 | 0.623 | 0.761 | 0.451 |
| P |  | 1.040 | 0.473 | 0.779 | 0.303 |
| Q |  | 1.076 | 0.673 | 0.961 | 0.496 |
| R |  | 1.163 | 0.547 | 0.669 | 0.379 |
| S |  | 1.035 | 0.453 | 0.618 | 0.348 |
| T |  | 0.938 | 0.347 | 0.397 | 0.413 |
| V |  | 1.268 | 0.711 | 0.747 | 0.699 |
| W |  | 0.884 | 0.407 | 0.418 | 0.283 |
| Y |  | 0.101 | 0.046 | 0.064 | 0.030 |
|  |  |  |  |  |  |

P) Standard deviation of secondary structure prediction for HMM method using Five-fold cross-validation, with the consideration of predicted three-state RSA information.

|  |  | Thresholds | | | |
| --- | --- | --- | --- | --- | --- |
|  |  | [4,16] | [9,16] | [9,36] | [16,36] |
| Total |  | 1.355 | 0.526 | 0.926 | 0.724 |
|  |  |  |  |  |  |
| A |  | 2.438 | 0.351 | 0.712 | 2.854 |
| C |  | 1.652 | 0.243 | 0.560 | 0.427 |
| D |  | 1.777 | 0.339 | 0.564 | 0.256 |
| E |  | 1.339 | 0.299 | 0.567 | 0.430 |
| F |  | 1.609 | 0.276 | 0.591 | 0.486 |
| G |  | 2.151 | 0.720 | 0.643 | 0.454 |
| H |  | 1.296 | 0.362 | 0.413 | 0.253 |
| I |  | 2.106 | 0.354 | 0.775 | 0.510 |
| K |  | 1.768 | 0.280 | 0.503 | 0.374 |
| L |  | 1.400 | 0.522 | 0.786 | 0.323 |
| M |  | 1.759 | 0.276 | 0.586 | 0.564 |
| N |  | 2.095 | 0.273 | 0.607 | 0.629 |
| P |  | 2.115 | 0.312 | 0.468 | 0.484 |
| Q |  | 1.785 | 0.426 | 0.356 | 0.578 |
| R |  | 1.928 | 0.350 | 0.723 | 0.376 |
| S |  | 1.363 | 0.264 | 0.493 | 0.395 |
| T |  | 1.488 | 0.359 | 0.707 | 0.282 |
| V |  | 1.509 | 0.496 | 0.394 | 0.491 |
| W |  | 1.670 | 0.361 | 0.272 | 0.306 |
| Y |  | 0.169 | 0.028 | 0.058 | 0.042 |
|  |  |  |  |  |  |

Q) Standard deviation of secondary structure prediction for HMM method using Five-fold cross-validation, with the consideration of residue-specific classification of actual RSA information.

|  |  | Thresholds | | | |
| --- | --- | --- | --- | --- | --- |
|  |  | Tertiles | Mean±SD | Mean | Median |
| Total |  | 0.854 | 0.485 | 1.169 | 0.327 |
|  |  |  |  |  |  |
| A |  | 0.803 | 0.554 | 1.050 | 0.485 |
| C |  | 0.690 | 0.419 | 0.751 | 0.382 |
| D |  | 1.024 | 0.521 | 1.107 | 0.305 |
| E |  | 0.959 | 0.475 | 1.096 | 0.441 |
| F |  | 1.115 | 0.531 | 1.210 | 0.273 |
| G |  | 1.042 | 0.493 | 1.218 | 0.417 |
| H |  | 0.705 | 0.393 | 0.720 | 0.558 |
| I |  | 1.190 | 0.315 | 1.338 | 0.337 |
| K |  | 0.773 | 0.331 | 0.901 | 0.373 |
| L |  | 0.589 | 0.351 | 0.582 | 0.434 |
| M |  | 0.678 | 0.617 | 0.854 | 0.364 |
| N |  | 1.122 | 0.485 | 1.320 | 0.380 |
| P |  | 0.947 | 0.515 | 1.053 | 0.288 |
| Q |  | 1.174 | 0.390 | 1.190 | 0.286 |
| R |  | 1.025 | 0.384 | 1.095 | 0.229 |
| S |  | 0.746 | 0.289 | 0.795 | 0.238 |
| T |  | 0.841 | 0.384 | 0.950 | 0.927 |
| V |  | 1.060 | 0.789 | 0.976 | 0.358 |
| W |  | 0.889 | 0.351 | 0.916 | 0.757 |
| Y |  | 0.090 | 0.042 | 0.107 | 0.021 |
|  |  |  |  |  |  |

R) Standard deviation of secondary structure prediction for HMM method using Five-fold cross-validation, with the consideration of residue-specific classification of predicted RSA information.

|  |  | Thresholds | | | |
| --- | --- | --- | --- | --- | --- |
|  |  | Tertiles | Mean±SD | Mean | Median |
| Total |  | 0.442 | 1.904 | 0.861 | 0.687 |
|  |  |  |  |  |  |
| A |  | 0.460 | 1.903 | 0.726 | 0.399 |
| C |  | 2.625 | 1.838 | 0.440 | 0.278 |
| D |  | 0.274 | 2.046 | 0.881 | 0.509 |
| E |  | 0.542 | 1.806 | 1.004 | 0.418 |
| F |  | 0.244 | 2.103 | 0.928 | 0.521 |
| G |  | 0.379 | 2.194 | 0.919 | 0.402 |
| H |  | 0.555 | 1.647 | 0.628 | 0.445 |
| I |  | 0.422 | 2.417 | 1.029 | 0.534 |
| K |  | 0.301 | 1.889 | 0.770 | 0.391 |
| L |  | 0.497 | 1.613 | 0.527 | 0.641 |
| M |  | 0.276 | 1.941 | 0.799 | 0.530 |
| N |  | 0.358 | 2.379 | 0.968 | 0.762 |
| P |  | 0.451 | 2.349 | 0.727 | 0.547 |
| Q |  | 0.546 | 2.099 | 1.079 | 0.410 |
| R |  | 0.525 | 2.140 | 0.887 | 0.476 |
| S |  | 0.262 | 1.594 | 0.830 | 0.442 |
| T |  | 0.597 | 1.712 | 0.690 | 0.423 |
| V |  | 0.532 | 1.933 | 0.996 | 0.692 |
| W |  | 0.234 | 1.825 | 0.883 | 0.312 |
| Y |  | 0.020 | 0.202 | 0.085 | 0.046 |
|  |  |  |  |  |  |
