# Supplementary material for: Impact of residue accessible surface area on the prediction of protein secondary structures
Source: BMC Bioinformatics. 2008 Aug 31;9:357. doi: 10.1186/1471-2105-9-357 (PMC2553345; doi:10.1186/1471-2105-9-357)
Supplement: Additional file 6 — Applied residue-specific thresholds used for classification of RSA values. [file 1471-2105-9-357-S6.doc]

Applied residue-specific thresholds used for classification of RSA values.

|  |  | Thresholds | | | | | | |
| --- | --- | --- | --- | --- | --- | --- | --- | --- |
|  |  | Mean | Median | Mean±SD | | Tertiles | | |
|  |  |  |  | Lower | Upper | Lower | | Upper |
| A |  | 0.188 | 0.072 | 0.000 | 0.418 | 0.016 | 0.224 | |
| C |  | 0.087 | 0.031 | 0.000 | 0.219 | 0.000 | 0.078 | |
| D |  | 0.392 | 0.382 | 0.121 | 0.663 | 0.223 | 0.510 | |
| E |  | 0.441 | 0.453 | 0.174 | 0.707 | 0.305 | 0.563 | |
| F |  | 0.108 | 0.043 | 0.000 | 0.263 | 0.013 | 0.098 | |
| G |  | 0.276 | 0.200 | 0.000 | 0.552 | 0.056 | 0.367 | |
| H |  | 0.248 | 0.203 | 0.031 | 0.465 | 0.099 | 0.312 | |
| I |  | 0.102 | 0.030 | 0.000 | 0.256 | 0.005 | 0.086 | |
| K |  | 0.460 | 0.463 | 0.235 | 0.685 | 0.370 | 0.551 | |
| L |  | 0.101 | 0.038 | 0.000 | 0.242 | 0.008 | 0.100 | |
| M |  | 0.145 | 0.055 | 0.000 | 0.342 | 0.009 | 0.157 | |
| N |  | 0.347 | 0.315 | 0.079 | 0.616 | 0.161 | 0.470 | |
| P |  | 0.291 | 0.258 | 0.053 | 0.529 | 0.123 | 0.394 | |
| Q |  | 0.364 | 0.358 | 0.106 | 0.623 | 0.195 | 0.484 | |
| R |  | 0.336 | 0.308 | 0.106 | 0.565 | 0.202 | 0.425 | |
| S |  | 0.276 | 0.218 | 0.016 | 0.537 | 0.075 | 0.376 | |
| T |  | 0.265 | 0.203 | 0.020 | 0.509 | 0.085 | 0.359 | |
| V |  | 0.110 | 0.035 | 0.000 | 0.270 | 0.006 | 0.100 | |
| W |  | 0.128 | 0.085 | 0.000 | 0.277 | 0.040 | 0.140 | |
| Y |  | 0.157 | 0.099 | 0.000 | 0.334 | 0.045 | 0.181 | |
|  |  |  |  |  | |  | | |
