# Supplementary material for: Impact of residue accessible surface area on the prediction of protein secondary structures
Source: BMC Bioinformatics. 2008 Aug 31;9:357. doi: 10.1186/1471-2105-9-357 (PMC2553345; doi:10.1186/1471-2105-9-357)
Supplement: Additional file 7 — Accuracy of secondary structure prediction for GOR, Chou-Fasman and HMM methods, with the consideration of random two- and three-state classification of actual RSA information. [file 1471-2105-9-357-S7.doc]

A) Accuracy of secondary structure prediction for GOR, Chou-Fasman and HMM methods using leave-one-out cross-validation, with the consideration of random two-state classification of actual RSA information. The presented data are averaged for 100 simulations. Totally, 1571044 residues were present in the assessed dataset. For more details, see the text.

|  |  | Method | | |
| --- | --- | --- | --- | --- |
|  |  | GOR | Chou-Fasman | HMM |
|  |  |  |  |  |
| Correct |  | 819656.62 | 730704.14 | 590231.26 |
| False |  | 751387.38 | 840339.86 | 980812.74 |
| Q3 |  | 51.952 | 47.844 | 37.671 |
| SD |  | 10.062 | 9.428 | 19.862 |
|  |  |  |  |  |
| A |  | 56.000 | 52.210 | 52.303 |
| C |  | 40.939 | 43.173 | 30.995 |
| D |  | 54.458 | 44.395 | 32.770 |
| E |  | 54.622 | 51.160 | 51.718 |
| F |  | 46.860 | 44.441 | 37.192 |
| G |  | 66.583 | 44.263 | 18.180 |
| H |  | 45.169 | 43.336 | 35.434 |
| I |  | 40.683 | 47.740 | 37.498 |
| K |  | 54.192 | 45.967 | 43.338 |
| L |  | 48.756 | 47.928 | 47.981 |
| M |  | 47.779 | 44.803 | 44.690 |
| N |  | 57.117 | 44.208 | 28.651 |
| P |  | 69.079 | 46.834 | 20.865 |
| Q |  | 52.364 | 48.040 | 47.533 |
| R |  | 50.367 | 46.130 | 44.465 |
| S |  | 49.687 | 43.733 | 31.133 |
| T |  | 44.905 | 41.278 | 28.638 |
| V |  | 51.271 | 49.733 | 31.927 |
| W |  | 43.722 | 42.550 | 40.070 |
| Y |  | 40.955 | 43.381 | 36.282 |
|  |  |  |  |  |

B) Accuracy of secondary structure prediction for GOR, Chou-Fasman and HMM methods using leave-one-out cross-validation, with the consideration of random three-state classification of actual RSA information.

|  |  | Method | | |
| --- | --- | --- | --- | --- |
|  |  | GOR | Chou-Fasman | HMM |
|  |  |  |  |  |
| Correct |  | 822448.1 | 733295.57 | 590026.08 |
| False |  | 748595.9 | 837748.43 | 981017.92 |
| Q3 |  | 52.203 | 48.098 | 37.657 |
| SD |  | 10.174 | 9.550 | 19.861 |
|  |  |  |  |  |
| A |  | 55.134 | 52.371 | 52.292 |
| C |  | 42.422 | 43.287 | 30.982 |
| D |  | 54.490 | 44.362 | 32.762 |
| E |  | 55.227 | 51.534 | 51.705 |
| F |  | 44.165 | 44.550 | 37.174 |
| G |  | 66.582 | 44.291 | 18.167 |
| H |  | 46.547 | 43.288 | 35.425 |
| I |  | 41.193 | 47.902 | 37.490 |
| K |  | 53.820 | 46.311 | 43.312 |
| L |  | 48.974 | 48.005 | 47.971 |
| M |  | 47.815 | 44.733 | 44.681 |
| N |  | 57.187 | 44.233 | 28.639 |
| P |  | 69.005 | 47.008 | 20.841 |
| Q |  | 52.502 | 48.320 | 47.519 |
| R |  | 50.062 | 46.330 | 44.453 |
| S |  | 49.675 | 43.845 | 31.117 |
| T |  | 45.268 | 41.742 | 28.628 |
| V |  | 52.963 | 49.957 | 31.918 |
| W |  | 43.893 | 42.767 | 40.054 |
| Y |  | 43.737 | 43.556 | 36.268 |
|  |  |  |  |  |
