# Supplementary material for: Impact of residue accessible surface area on the prediction of protein secondary structures
Source: BMC Bioinformatics. 2008 Aug 31;9:357. doi: 10.1186/1471-2105-9-357 (PMC2553345; doi:10.1186/1471-2105-9-357)
Supplement: Supplementary file 7 [file 1471-2105-9-357-S5.doc]

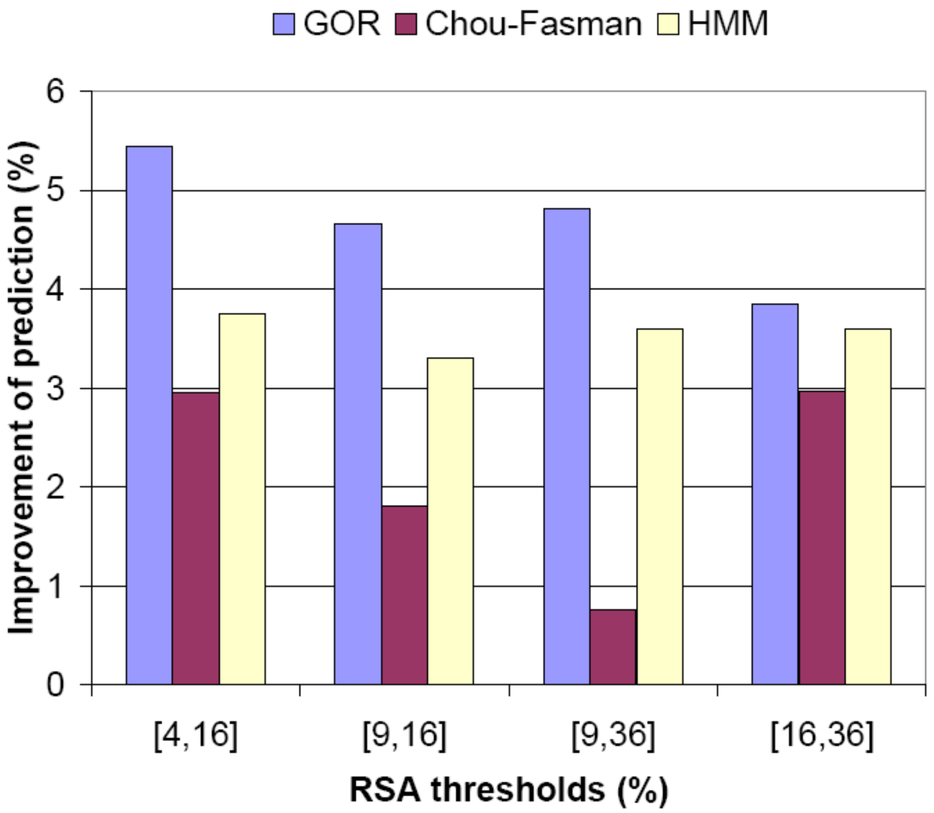

GOR
Chou-Fasman
HMM
Improvement of prediction (%)
0
1
2
3
4
5
6
[4,16]
[9,16]
[9,36]
[16,36]
RSA thresholds (%)
